# Risky-choice framing effects persist when option descriptions are matched and complete: A replication and extension of DeKay and Dou (2024)

**DOI:** 10.3758/s13423-025-02771-w

**Published:** 2026-04-14

**Authors:** Michael L. DeKay

**Affiliations:** Department of Psychology, The Ohio State University, 224 Lazenby Hall, 1827 Neil Avenue, Columbus, OH 43210 USA

**Keywords:** Choice, Decision making, Risk, Explicated valence account, Fuzzy-trace theory

## Abstract

People’s choices involving money, health, and other matters can often be steered toward safer or riskier options by framing the options as gains or losses. This risky-choice framing effect is well established, but its direction and magnitude depend on whether the options are described completely or incompletely and whether the descriptions are matched or mismatched (in terms of good and bad outcomes) in the gain and loss frames. In a recent experiment involving 18 combinations of option descriptions (nine in each frame), DeKay and Dou, *Psychological Science*, *35*(8), 918–932 (2024) found that the valence or gist of the descriptions accounted for substantial variation in risk preferences, favoring categorical theories of framing over more mathematical theories. However, a residual framing effect and a framing effect in choices between completely described options contradicted the categorical theories and previous results. The effect for completely described options was especially surprising, but it was based on data from only 97 participants. The experiment reported here replicated all these effects in a large sample ($N = 1,697$) well matched to US Census data and with many more participants (477) making choices between completely described options. Risky-choice framing effects do stem partly from mismatched option descriptions, but a framing effect persists even when descriptions are matched and complete. The latter results expand the effect’s relevance to situations where it was thought to be absent. Among several theory specifications, only a new rescaled version of the explicated valence account explains these effects in the replication and in DeKay and Dou’s online and student samples.

**Supplementary Information:**

The online version contains supplementary material available at 10.3758/s13423-025-02771-w.

## Introduction

Before I got married I had six theories about bringing up children; now, I have six children and no theories.

—John Wilmot, perhaps^1^

Data, like children, have a way of challenging theories. That is good; theories should be challenged. The same goes for empirical findings, which need to be challenged to establish their replicability and generalizability, or lack thereof.

The research reported here replicates and extends an intriguing collection of findings from DeKay and Dou (2024; hereafter DD) that challenge the usual explanations of the well-known risky-choice framing effect. I begin with the obligatory description of Tversky and Kahneman’s (1981) classic problem, in which 600 people are expected to die from an unusual disease, and one must choose between a certain option and an all-or-none risky option with the same expected value (EV). In the gain frame, the certain option provides that “200 people will be saved,” while the risky option provides “a 1/3 probability that 600 people will be saved and a 2/3 probability that no people will be saved.” In the loss frame, the certain option provides that “400 people will die,” while the risky option provides “a 1/3 probability that no people will die and a 2/3 probability that 600 people will die.” The typical result — that people tend to choose the certain option in gains and the risky option in losses — is among the most well-established findings in psychology (DeKay et al., 2022; Im & Chen, 2022; Klein et al., 2014; Kühberger, 1998; Kühberger et al., 1999; Levin et al., 1998; Piñon & Gambara, 2005; Steiger & Kühberger, 2018).

### Variants of risky-choice framing problems

Interestingly, the robustness of the framing effect in problems like the one above depends on the descriptions of the certain options being incomplete. In gains, the description specifies what happens to 200 people, but not the other 400; in losses, the situation is reversed. Researchers noting this inconsistency have created numerous problem variants involving different combinations of complete and incomplete option descriptions. Depending on the details, the framing effect can be amplified, eliminated, or reversed (for a review, see Broniatowski & Reyna, 2018).

To facilitate discussion of such variants, DD proposed the framework in Table 1. The description of the certain option can include only the good aspect (e.g., “200 people will be saved”), only the bad aspect (e.g., “400 people will not be saved”), or both. The same is true for the risky option, leading to nine combinations of descriptions in each frame (cells G1 to G9 and L1 to L9; ignore the scores in these cells for now). The standard framing effect involves cells G4 and L6, which are *mismatched* in the sense that the descriptions include different aspects in gains and losses. Comparisons involving the same aspects in both frames (e.g., G1 vs. L1) are *matched*.^2^

**Table 1:** Possible combinations of good and bad aspects of options in a risky-choice framing problem with 600 lives at risk

	Description and valence of the certain option		
Description and valence of the risky option	Good aspect only (+1)	Both aspects (0)	Bad aspect only (−1)
Gain frame			
	200 people are saved	200 people are saved and 400 people are not saved	400 people are not saved
Good aspect only (+1)	G1 (0)	G2 (+1)	G3 (+2)
1/3 chance that 600 people are saved			
Both aspects (0)	G4 (−1)	G5 (0)	G6 (+1)
1/3 chance that 600 people are saved and	Standard FE	Complete info.	
2/3 chance that no people are saved			
Bad aspect only (−1)	G7 (−2)	G8 (−1)	G9 (0)
2/3 chance that no people are saved			
Loss frame			
	200 people do not die	200 people do not die and 400 people die	400 people die
Good aspect only (+1)	L1 (0)	L2 (+1)	L3 (+2)
1/3 chance that no people die			
Both aspects (0)	L4 (−1)	L5 (0)	L6 (+1)
1/3 chance that no people die and		Complete info.	Standard FE
2/3 chance that 600 people die			
Bad aspect only (−1)	L7 (−2)	L8 (−1)	L9 (0)
2/3 chance that 600 people die			

FE* =* framing effect. Option descriptions are shortened to save space. In cells G1 to G9 and L1 to L9, the number in parentheses is the difference between the valence of the risky option and the valence of the certain option. Higher valence differences indicate stronger predicted preferences for the risky option. Predictions are from DeKay and Dou’s (2024) interval-scaled version of the explicated valence account. Predictions from alternative models appear in the Method section. Adapted from “Risky-choice framing effects result partly from mismatched option descriptions in gains and losses,” by M. L. DeKay and S. Dou, 2024, *Psychological Science*, *35*(8), p. 920. (10.1177/09567976241249183). Copyright 2024 by the authors. Adapted with permission

When the certain and risky options are described completely (G5 vs. L5, a matched comparison), the framing effect is typically eliminated (Betsch & Kraus, 1999; De Boeck et al., 2022; DeKay et al., 2022; Kühberger, 1995; Mandel, 2014; Schulte-Mecklenbeck & Kühberger, 2014; Stocké, 1998; Tombu & Mandel, 2015). Most other comparisons have been mismatched. For example, the framing effect is reversed when the certain options are described only by negations (G6 vs. L4; Kühberger, 1995). With the usual (incomplete) certain options, the framing effect is typically eliminated when the zero components of the risky options are omitted (G1 vs. L9) and amplified when the nonzero components are omitted (G7 vs. L3; Chick et al., 2016; Kühberger & Tanner, 2010; Reyna & Brainerd, 1991, 2011; Reyna et al., 2014, 2021; Zhou et al., 2021). Other variants have also been studied.^3^

### Theoretical accounts

#### Prospect theory

According to prospect theory (PT; Kahneman & Tversky, 1979; Tversky & Kahneman, 1981), the wording of the outcomes in the original disease problem (*saved* vs. *die*) leads participants to adopt different reference points in the two frames. The framing effect is then predicted by PT’s S-shaped value function (concave for gains, convex for losses), its inverse-S-shaped weighting function (overweighting of low-probability outcomes, underweighting of moderate- and high-probability outcomes), or their multiplicative combination (see DeKay & Garge, 2023, for details).

The results for framing-problem variants challenge PT because the theory does not specify how negations like “400 people will not be saved”^4^ should be handled or what, if anything, participants should infer when option descriptions are incomplete (Kühberger & Tanner, 2010; Mandel, 2001). Other mathematical theories of risky choice (e.g., Birnbaum’s (2008) transfer-of-attention-exchange theory; Brandstätter et al.’s (2006) priority heuristic) are similarly underspecified.

#### Fuzzy-trace theory

Fuzzy-trace theory (FTT; Broniatowski & Reyna, 2018; Reyna, 2012; Reyna & Brainerd, 1995) relies heavily on gist representations, which capture the essential meaning of information simply and qualitatively. FTT extended the gist concept from psycholinguistics to numerical information, with attention to people’s basic numerical abilities and representations of quantities. In the gain frame of the disease problem, the categorical gist of the certain option is “some people will be saved,” and that of the risky option is “some people will be saved or no people will be saved.” The distinction between *some saved* and *none saved* favors the certain option. In losses, the comparison between *some die* and *none die* favors the risky option. FTT also includes ordinal gist representations and interval (verbatim) representations, but these rarely matter for framing problems because the options generally have equal EVs.

Several comparisons of FTT and PT have relied on “critical tests” involving incomplete descriptions of the risky options (Chick et al., 2016; Reyna, 2012; Reyna & Brainerd, 1995; Reyna et al., 2014, 2021). According to FTT, omitting risky options’ zero components (G1 vs. L9) eliminates the framing effect because the categorical *some* versus *none* comparisons are absent, whereas omitting the nonzero components (G7 vs. L3) amplifies the effect because those comparisons are highlighted. Broniatowski and Reyna (2018) specified how FTT deals with negations, allowing the theory to account for additional results. However, those authors did not indicate how comparisons between negations (e.g., “400 people will not be saved”) and zero outcomes (e.g., “no people will be saved”) are handled, so the theory does not always make clear predictions. Building on DD, I consider three FTT specifications to address this issue.^5^

#### The explicated valence account

Mandel (2001) differentiated descriptor formulations (e.g., *saved* vs. *die*) from outcome formulations (whether the outcomes themselves have positive or negative valence), showing that neither influences choice when they are unconfounded. Following additional evidence from Kühberger and Gradl (2013) regarding options with positive, negative, or mixed valence, Tombu and Mandel (2015) proposed the explicated valence account (EVA). In the gain frame of the disease problem, the certain option’s positive valence is preferred to the risky option’s mixed valence. In losses, the risky option’s mixed valence is preferred to the certain option’s negative valence.^6^ These categorical comparisons account for the results of many variants, but EVA requires further specification for others. For example, it does not obviously predict a larger framing effect for the positive-versus-negative valence comparisons in G7 versus L3. Like DD, I consider three EVA specifications to address this issue. In addition, a fourth specification distinguishes between similarly valenced negations and zero outcomes (e.g., “400 people will not be saved” and “no people will be saved”).

#### The lower-bounding hypothesis

Numerous authors have noted that incompletely described options are ambiguous (Fisher & Mandel, 2021; Kühberger & Tanner, 2010; Mandel, 2001, 2014; Okder, 2012; Stocké, 1998). In the disease problem, “200 people will be saved” might be interpreted as “*at least* 200 people will be saved” for logical reasons (e.g., some of the others might also be saved) or closely related linguistic reasons (see Mandel, 2014, for details). Similarly, “400 people will die” might be interpreted as “*at least* 400 people will die.” If there are no analogous effects for completely described risky options, the apparent EV differences between the certain and risky options can explain the original framing effect (note that, in contrast to FTT and EVA, the decision-relevant representation is not categorical). This account is called the lower-bounding hypothesis (LBH; Fisher, 2022; Fisher & Mandel, 2021; Mandel & Kelly, 2024).^7^

Mandel (2014) assessed LBH for three pairs of cells in Table 1 (G4 vs. L6, G5 vs. L5, and G2 vs. L8). Lower-bound interpretations were most common for incompletely described certain options and (to a lesser extent) incompletely described risky options.^8^ Applying LBH to solitary negations (e.g., “*at least* 400 people will not be saved”) and solitary zero outcomes (e.g., “*at least* a 2/3 probability that no people will be saved”) seems straightforward, but there are multiple possibilities for predicting preferences. I consider three LBH specifications, but the simplest two are indistinguishable from DD’s EVA specifications.

The LBH explanation of the original framing effect hinges on the ambiguity of option descriptions being asymmetric in gains and losses (Mandel, 2001, 2014). Strategies for eliminating ambiguity have included using “exactly” in the option descriptions (Mandel, 2014; Simmons & Nelson, 2013; see Mandel, 2013/2020, for discussion), providing causal explanations that facilitate inferences about missing information (Jou et al., 1996), and training participants to complete any incomplete option descriptions (Chick et al., 2016; Reyna et al., 2021). In contrast, eliminating asymmetry means using a matched set of cells from Table 1. Mandel (2001, Table 3) used cells G1, G9, L1, and L9, whereas DD used all 18 cells. Using completely described options (G5 vs. L5) eliminates the ambiguity and the asymmetry. Overall, results indicate that asymmetric ambiguity may be a sufficient condition for framing effects, but it does not appear to be necessary.

### DeKay and Dou’s (2024) model and experiment

DD contributed to this line of research by organizing previous findings within the framework of Table 1, conducting a large experiment with all combinations of options in that framework, and evaluating the performance of different versions of EVA and FTT in predicting the results.

#### Their model

In DD’s preferred model, dubbed “interval-scaled EVA,” the valence of an option description is coded +1 if it includes only the good aspect, −1 if it includes only the bad aspect, and 0 if it includes both. Preference for the risky option in any choice is predicted by *ValenceDiff* = *Valence*_Risky_ − *Valence*_Certain_. These differences, which appear in the cells of Table 1, range from −2 to +2. For any pair of gain and loss cells, the framing effect is predicted by the difference between *ValenceDiff* scores. DD used *FE*_Pred_ = (*ValenceDiff*_Losses_ − *ValenceDiff*_Gains_) ∕2, which yields +1 for the standard framing effect (hereafter denoted L6–G4 to convey the direction of subtraction). This model accounts for results in all seven framing-effect variants in Broniatowski and Reyna’s (2018) review.

#### Their experiment

DD investigated all cells of Table 1 using 906 CloudResearch participants and 521 undergraduates, each of whom made four choices. Consistent with past results, framing effects ranged from reversed to amplified, depending on how gain and loss cells were paired. In both samples, choice proportions were well predicted by interval-scaled EVA, two other EVA specifications, and two FTT specifications, with interval-scaled EVA outperforming the other models in terms of the Bayesian information criterion (BIC). Regardless of the model, however, there was always a residual framing effect; previous research suggested no residual effect. Moreover, in DD’s online and combined samples, the framing effect was significant even when both options were completely described, contradicting EVA, FTT, and previous results.

#### Why replicate?

I replicated DD’s experiment for three reasons. First, results from convenience samples might not generalize to any identifiable population, such as adults in the USA (but see Coppock et al., 2018). Second, the residual framing effect was at odds with theory and previous data. The significant effect in choices between completely described options was particularly suspect, as it was based on only 97 participants. Third, DD always presented choices with the certain option listed first, which can sometimes yield larger framing effects (Kühberger & Gradl, 2013). The replication addressed all three concerns. An additional goal for this article is to glean further insights from comparisons of competing theoretical accounts.

## Method

### Participants

In the *Design* section below, I justify the target sample size of 1,680 participants after exclusions. In spring 2024, I used CloudResearch Connect to recruit 1,943 participants for an incentive of $0.60.^9^ I chose Connect because its quota-sampling option provided an excellent combination of data quality and representativeness in a recent comparison of nine opt-in nonprobability sources (Stagnaro et al., 2024). In the same comparison, Connect yielded a risky-choice framing effect that was very near the global effect size for all nine samples.

I used demographic targeting to match the most recent US Census data for adults on gender (man or woman, within 0.3 percentage points; the census does not have other categories), race (White, Black or African American, or another race, within 0.3 pp), ethnicity (Hispanic or not, within 2.0 pp), geographic region (Midwest, Northeast, South, or West, within 1.8 pp), and personal income (three categories, within 3.0 pp). Because of the limitations of Connect’s participant pool for large samples, the initial sample underrepresented persons aged 60 years or older by 6.1 pp (24.6% instead of 30.7%; the target in Connect was 26.0%), but the sample was within 2.4 pp for the other three age categories. I was unable to match the census data for education: Those with a high-school diploma or less were underrepresented (19.9% instead of 38.8%), while those with a bachelor’s degree or higher were overrepresented (48.2% instead of 34.8%). See the Online Supplemental Material (OSM) for details.

Following DD, I excluded participants who were flagged by Qualtrics as fraudulent or duplicates (94), as well as those who finished the task too quickly (15) or too slowly (17), skipped more than one of the eight choice and rating questions (40), or answered any pair of choice and rating questions inconsistently (80; see the OSM). Participants in the final sample ($N = 1,697$) were from all 50 states and the District of Columbia and were 18–86 years old (*M*=45.4, *SD*=15.5); 48.0% identified as a man and 52.0% as a woman; 77.4% identified as White, 12.2% as Black or African American, 6.7% as Asian or Pacific Islander, and 3.7% as another race; 15.0% also identified as Hispanic. Except for the highest age bracket ($\ge 60\ \mathrm{years}$) and education, the final sample remained a good match to the census data.

### Procedure

The procedure was nearly identical to that in DD. Participants responded to four randomly ordered scenarios, each in a different domain (e.g., disease, investment) but always in the same frame (gains or losses). In each scenario, participants chose between a certain option and an all-or-none risky option with the same EV and indicated their strength of preference on a 7-point scale with endpoints labeled *strong preference for Program A* [or *B*] and with the midpoint labeled *no preference at all*. Which program was listed first and called Program A was varied randomly for each scenario for each participant (DD always listed the certain option first).

The scenarios involved an infectious disease with 3,000 people at risk, a drought with 24,000 acres of crops at risk, a financial investment with $15,000 at risk, and a wildfire with 7,200 animals at risk (the latter three were based on Peters & Levin, 2008). The value and wording of the probability of the better outcome in the risky option varied across domains (“a 2/5 probability,” “a 50% chance,” “a one out of three chance,” and “a one-fourth probability,” respectively).

At the end of the survey, participants answered one debriefing question. Connect provided demographic information for all participants.

### Design

Each participant was assigned to one of the 18 cells in Table 1. In ten of the cells, the description of the certain option or the risky option included both good and bad aspects, which could be mentioned in either order. I used both orders, which yielded 28 conditions in the experiment. Like DD, I omitted the possible mixed orders in cells G5 and L5, where the certain and risky options could be described in different orders in the same problem.

Compared to DD, I increased the minimum sample-size target to 60 participants per cell so that each matched comparison (e.g., L1–G1) would involve at least 120 participants and 480 choices. I oversampled cells G3, G7, L3, and L7 (120 participants each) because those cells were likely to generate the most extreme preferences for the certain or risky options. I oversampled cells G5 and L5 to an even greater extent (240 participants each) to have ample power for detecting a small effect ($d = 0.2$) in the complete-information comparison. The total target sample size was thus 1,680. I collected more data to allow for exclusions (see Footnote 9).

For power analyses, I used Kumle et al.’s (2021) simulation procedures for mixed-effects models (see the OSM). With $n = 480$ for the complete-information comparison (L5–G5), estimated power for a small effect was .84.^10^ With $n = 120$, as in most other matched comparisons, power was .74 for a medium effect ($d = 0.5$) but only .20 for a small effect.^11^ Note, however, that tests comparing only two cells would involve as little as 7% of the data. In the primary model for predicting participants’ choices in the full data set (target $N = 1,680$), power was .997 for a very small effect ($d = 0.1$) of a 1-unit change in *ValenceDiff* and .98 for a small residual effect of *Frame* (see Footnote 10). The most important power results were those for the complete-information comparison and the primary model.

### Theory predictions

Table 2 shows predicted preferences from three FTT specifications, four EVA specifications, and one LBH specification for all 18 cells of Table 1. Five of the eight specifications are from DD; those five plus the LBH specification were preregistered. Table 3 shows the predicted framing effects (*FE*_Pred_ values) from the same specifications for nine matched cell pairs.

**Table 2:** Predicted preferences for the risky option from several specifications of FTT, EVA, and LBH for variants of a risky-choice framing problem with 600 lives at risk

Cell	Certain option	Risky option	FTT1	FTT2	FTT3 (new)^a^	Interval-scaled EVA	Original EVA	Free-valence-differences EVA^b^	Rescaled EVA (new)^a^	Separate-effects LBH (new)^c^
Gain frame										
G1	200 saved	1/3 chance 600 saved	0	0	0	0	0	0, 0, 0	0	1, 1
G2	200 saved, 400 not saved	1/3 chance 600 saved	1	1	1	1	1	1, 0, 0	***f***	0, 1
G3	400 not saved	1/3 chance 600 saved	**1**	**1**	2	2	**1**	0, 0, **1**	**2** ***f***	−1, 1
G4	200 saved	1/3 chance 600 saved, 2/3 chance none saved	−1	−1	−1	−1	−1	−1, 0, 0	−1	1, 0
G5	200 saved, 400 not saved	1/3 chance 600 saved, 2/3 chance none saved	0	0	0	0	0	0, 0, 0	**−1 +** ***f***	0, 0
G6	400 not saved	1/3 chance 600 saved, 2/3 chance none saved	1	1	**0**	1	1	0, 1, 0	**−1 + 2** ***f***	−1, 0
G7	200 saved	2/3 chance none saved	−2	−2	−2	−2	**−1**	0, 0, **−1**	−2	1, −1
G8	200 saved, 400 not saved	2/3 chance none saved	**0**	−1	−1	−1	−1	0, −1, 0	**−2 +** ***f***	0, −1
G9	400 not saved	2/3 chance none saved	**1**	0	**−1**	0	0	0, 0, 0	**−2 + 2** ***f***	−1, −1
Loss frame										
L1	200 do not die	1/3 chance none die	**−1**	0	**1**	0	0	0, 0, 0	**2 − 2** ***f***	1, 1
L2	200 do not die, 400 die	1/3 chance none die	**0**	1	1	1	1	1, 0, 0	**2 −** ***f***	0, 1
L3	400 die	1/3 chance none die	2	2	2	2	**1**	0, 0, **1**	2	−1, 1
L4	200 do not die	1/3 chance none die, 2/3 chance 600 die	−1	−1	**0**	−1	−1	−1, 0, 0	**1 − 2** ***f***	1, 0
L5	200 do not die, 400 die	1/3 chance none die, 2/3 chance 600 die	0	0	0	0	0	0, 0, 0	**1 −** ***f***	0, 0
L6	400 die	1/3 chance none die, 2/3 chance 600 die	1	1	1	1	1	0, 1, 0	1	−1, 0
L7	200 do not die	2/3 chance 600 die	**−1**	**−1**	−2	−2	**−1**	0, 0, **−1**	**−2** ***f***	1, −1
L8	200 do not die, 400 die	2/3 chance 600 die	−1	−1	−1	−1	−1	0, −1, 0	**−** ***f***	0, −1
L9	400 die	2/3 chance 600 die	0	0	0	0	0	0, 0, 0	0	−1, −1

FTT* =* fuzzy-trace theory; EVA* =* explicated valence account; LBH* =* lower-bounding hypothesis; *f* = a scaling factor for negations. Option descriptions are shortened to save space. Higher numbers indicate stronger predicted preferences for the risky option. Boldface type indicates predicted preferences that differ from those of interval-scaled EVA, which was the best-performing specification in DeKay and Dou (2024). Adapted from “Risky-choice framing effects result partly from mismatched option descriptions in gains and losses,” by M. L. DeKay and S. Dou, 2024, *Psychological Science*, *35*(8), p. 921 (10.1177/09567976241249183). Copyright 2024 by the authors. Adapted with permission

^a^This theory specification was not preregistered. ^b^The three predicted-preference codes are for positive-versus-mixed, mixed-versus-negative, and positive-versus-negative valence differences, respectively. Each code has its own coefficient in models for predicting choice. ^c^The two codes are for certain and risky options, respectively. The predicted preference is the weighted difference between the risky- and certain-option codes, with each code having its own coefficient in models for predicting choice

**Table 3:** Predicted framing effects from several specifications of FTT, EVA, and LBH in nine matched cell pairs

Cell pair	FTT1	FTT2	FTT3 (new) ^a^	Interval-scaled EVA	Original EVA	Free-valence-differences EVA	Rescaled EVA (new) ^a^	Separate-effects LBH (new)
L1–G1	−0.5	0	0.5	0	0	0	1 − *f*	0
L2–G2	−0.5	0	0	0	0	0	1 − *f*	0
L3–G3	0.5	0.5	0	0	0	0	1 − *f*	0
L4–G4	0	0	0.5	0	0	0	1 − *f*	0
L5–G5	0	0	0	0	0	0	1 − *f*	0
L6–G6	0	0	0.5	0	0	0	1 − *f*	0
L7–G7	0.5	0.5	0	0	0	0	1 − *f*	0
L8–G8	−0.5	0	0	0	0	0	1 − *f*	0
L9–G9	−0.5	0	0.5	0	0	0	1 − *f*	0

FTT = fuzzy-trace theory; EVA = explicated valence account; LBH = lower-bounding hypothesis; *f* = a scaling factor for negations. Predicted framing effects (*FE*_Pred_ values) are half the difference between predicted preferences in losses and gains (see Table 2). For comparison, the predicted framing effect in the standard comparison (L6–G4) is 1

^a^This theory specification was not preregistered

#### Fuzzy-trace theory

In 10 of the 18 cells, the three FTT versions make the same predictions as interval-scaled EVA. In the remaining cells, the versions differ in how they handle categorical comparisons between “some people will not be saved” and “no people will be saved” in gains and between “some people will not die” and “no people will die” in losses. DD’s FTT1 is based on Broniatowski and Reyna (2018), who stated that in the gain frame, (a) *none saved* is a subset of *some saved* (an odd claim, in my opinion), and (b) *some saved* is categorically preferred to *some not saved*. Although those authors did not pursue the implications, these statements lead to the counterintuitive conclusion that *none saved* is preferred to *some not saved* in gains; similarly, *some do not die* is preferred to *none die* in losses. In contrast, DD’s FTT2 treats these categories as equally preferred or not comparable, so they never contribute to option preferences.

The third possibility, embodied in FTT3, is that the ordering is the opposite of that in FTT1 (e.g., in FTT3, *some not saved* is preferred to *none saved* in gains). This ordering is consistent with Kühberger and Tanner’s (2010, p. 320) opinion that “Intuitively, *some people will not be saved* seems to be a better outcome than *no one will be saved*; it certainly can’t be worse.”^12^ In accord with previous FTT explanations (Chick et al., 2016; Reyna, 2012; Reyna & Brainerd, 1995; Reyna et al., 2014, 2021), FTT3 also assumes that categorical preferences are amplified when the only comparison is between *some* and *none* (cells G7 and L3) or, by extension, between *some* and *not* (cells G3 and L7).^13^

The three FTT versions differ in their predictions for framing effects in most matched cell pairs, with FTT3 making more positive predictions than FTT1 or FTT2. However, none of the versions predicts a framing effect in choices between completely described options (L5–G5) because competing categorical comparisons involving other aspects of the options always lead to predictions of no preference in both gains (G5) and losses (L5).^14^

#### The explicated valence account

In addition to interval-scaled EVA, I consider three other specifications. In DD’s “original EVA,” which was intended to be consistent with Tombu and Mandel (2015), positive-versus-negative valence differences are treated the same as positive-versus-mixed and mixed-versus-negative valence differences, so values of $\pm$2 are replaced with $\pm$1. In DD’s “free-valence-differences EVA,” there are separate codes for the three types of valence difference, each with its own coefficient. These three EVA specifications predict no framing effects in matched cell pairs.

In the final EVA specification (explained in greater detail in the OSM), I incorporated Kühberger and Tanner’s (2010) intuition that negations in certain options (e.g., *some not saved*) may be evaluated as less extreme than zero outcomes in risky options (e.g., *none saved*; see Footnote 12). To do so, I rescaled the certain-option valences in Table 1 from (+1, 0 −1) to reduce the extremity of negations while keeping the valences of completely described options equal to the average of the valences of their good and bad aspects. Specifically, I used the scaling factor $f$ in the following linear transformations:

1 $${RValence}_{\mathrm{Certain}} = \left\{\begin{array}{ll}f\times{Valence}_{\mathrm{Certain}}+\left({1}-f\right) & \mathrm{ in \ gains}\\ f\times {Valence}_{\mathrm{Certain}}-\left({1}-f\right) & \mathrm{ in \ losses.}\end{array}\right.$$

For example, if $f = 2/3$, the original certain-option valences (+1, 0 −1) become (+1, +1/3, −1/3) in gains, with −1/3 for the negation (e.g., “400 people will not be saved”). In losses, the certain-option valences become (+1/3, −1/3, −1), with +1/3 for the negation (e.g., “200 people will not die”). The risky-option valences are not rescaled (i.e., $\mathit{RValence}_\mathrm{Risky} = \mathit{Valence}_\mathrm{Risky}$) because the descriptions do not include negations. In any particular problem, the valence difference in favor of the risky option is computed as before: ${RValenceDiff} = {RValence}_\mathrm{Risky}-{RValence}_\mathrm{Certain}$.

This EVA specification yields an interesting prediction^15^ for framing effects in matched comparisons. In those comparisons, the valences of the risky options cancel, as in the other EVA specifications, but the rescaled valences of the certain options do not. Specifically, the predicted framing effect in every matched comparison is:

2 $$\mathit{FE}_\mathrm{Pred} = \frac{\mathit{RValenceDiff}_\mathrm{Losses}-\mathit{RValenceDiff}_\mathrm{Gains}}2 = \frac{2\left(1-f\right)}2 = 1-f.$$

Continuing the example, if $f = 2/3$, the predicted framing effect in matched comparisons is 1/3 as large as the standard framing effect. In other words, allowing evaluations of negations in certain options to be less extreme than zero outcomes in risky options (i.e., $f < 1$) implies a smaller but nonzero framing effect in matched comparisons, including L5–G5. Rather than guess at the appropriate scaling factor, I estimate $f$ as an additional parameter in the *Results* section.

#### The lower-bounding hypothesis

To extend LBH to all cells of Table 1, I scored option descriptions as +1 if lower-bounding increases the EV and −1 if it decreases the EV. For example, “*at least* 200 people will be saved” is scored +1, while “*at least* 400 people will not be saved” is scored −1. The same reasoning applies to incompletely described risky options. For example, “*at least* a 1/3 probability that 600 people will be saved” is scored +1. Completely described options are scored zero because there is no ambiguity. Scores in losses are the same as those in gains.

If preferences are again determined by the difference between the scores for risky and certain options, the predictions are identical to those of interval-scaled EVA (the coding is identical to that in Table 1). If the magnitudes of the differences are ignored, so that differences of $\pm$2 are replaced with $\pm$1, the predictions are identical to those of original EVA. This correspondence between LBH and EVA is notable, especially since Tombu and Mandel (2015) did not mention LBH in their rationale for EVA. Of course, the equivalence of the predictions for the problems considered here does not imply the equivalence of the explanatory accounts.

The above versions of LBH assume that quantifier interpretations in the certain and risky options are equally important. Kühberger and Tanner (2010) argued that incompletely described risky options are more ambiguous than their certain-option counterparts because any distribution of lives saved or lost could occur with the unspecified probability (also see Tombu & Mandel, 2015, p. 466). If so, interpretations of risky options might be more important than those of certain options. On the other hand, Mandel (2014, Table 4) reported that noticeably fewer participants adopt lower-bound interpretations for incomplete risky options than for incomplete certain options, suggesting that interpretations of risky options might be less important. To investigate the issue, I preregistered a model with separate coefficients for certain and risky options, with the prediction (based on reanalysis of DD’s data) that the effect of risky options would be substantially larger than that of certain options. This “separate-effects LBH” model is like the rescaled EVA model in Eq. 1 but without the second term that distinguishes gains and losses. Like the other three EVA specifications, separate-effects LBH predicts no framing effects in matched cell pairs.

**Table 4:** Number and percentage of times participants chose the risky option in each cell

Gain frame			Loss frame		
Cell	Number	Percentage	Cell	Number	Percentage
G1	92/252	36.7	L1	117/224	52.2
G2	108/246	43.9	L2	158/246	64.2
G3	307/495	62.0	L3	413/502	82.3
**G4**	**59/248**	**23.8**	L4	61/239	25.5
G5	234/936	25.0	L5	336/962	34.9
G6	89/253	35.2	**L6**	**106/223**	**47.5**
G7	52/460	11.3	L7	109/526	20.7
G8	42/234	17.9	L8	68/240	28.3
G9	46/220	20.9	L9	74/250	29.6
Overall	1,029/3,343	30.8	Overall	1,442/3,412	42.3

Each participant made four choices in one of the 18 cells. Some cells were oversampled by design. Boldface type indicates cells involved in the standard framing effect

## Results

Table 4 reports how often participants chose the risky option in each cell. The percentages in gains and losses differed by 23.7 pp for the standard framing effect (L6–G4) and by 11.5 pp for the full experiment.

### Primary analysis using interval-scaled EVA

Following DD, I used mixed-effects logistic regression (run in lme4; Bates et al., 2015) to predict choice of the risky option. I included *ValenceDiff* from interval-scaled EVA, *Frame* ($\mathrm{gains} = -0.5$, $\mathrm{losses} = 0.5$), and their interaction as predictors, plus a random intercept for participants.

**Fig. 1:**
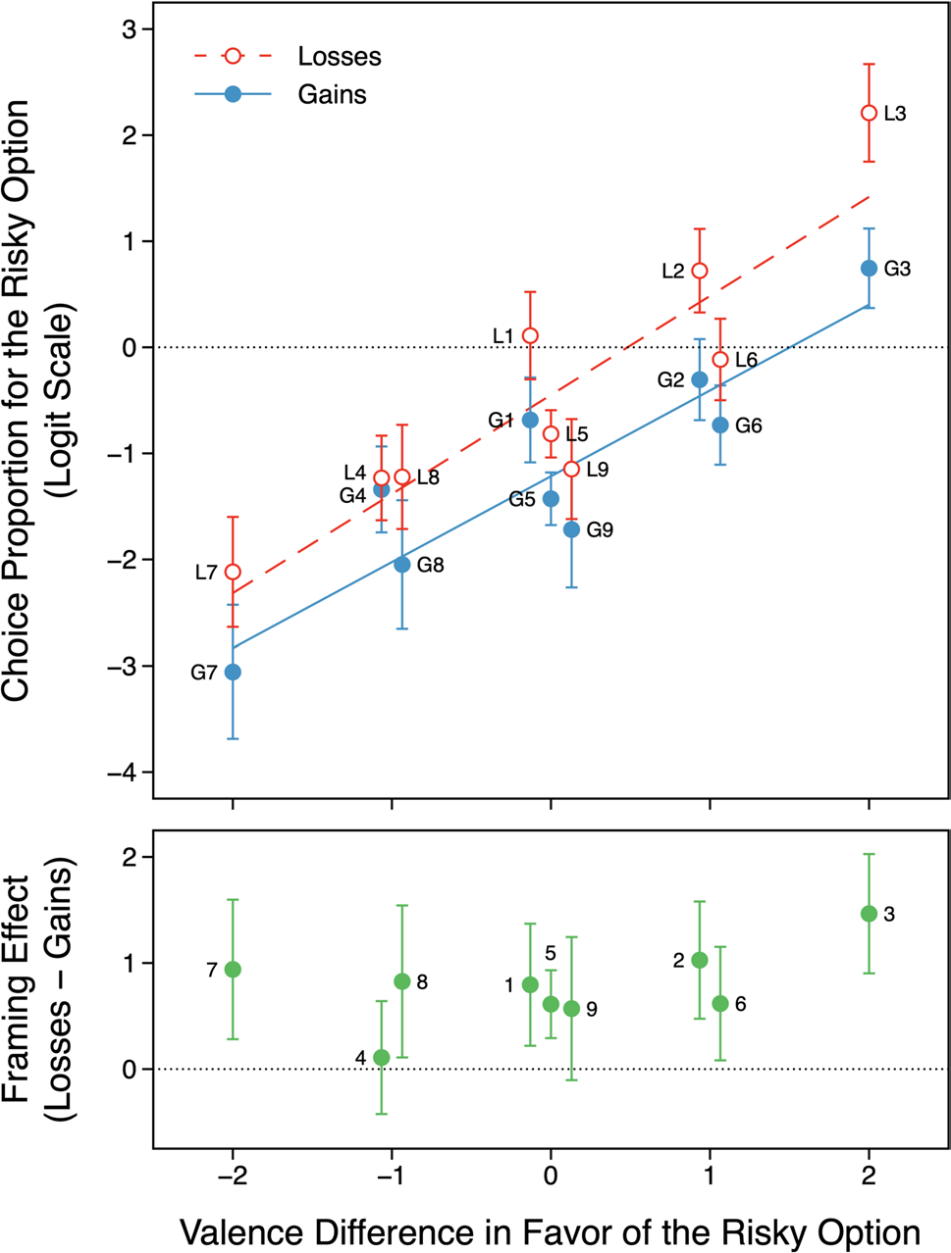
**Top:** Choice proportions for the risky option as a function of valence difference and frame. Valence differences on the horizontal axis are from DeKay and Dou’s (2024) interval-scaled version of the explicated valence account. Labels correspond to the cells in Table 1. Error bars indicate 95% confidence intervals. Regression lines are from the primary model. To reduce overlap, some points are shifted slightly to the left or right. **Bottom:** Framing effects in the nine corresponding matched comparisons (e.g., the leftmost point, labeled 7, is for L7–G7)

The results matched those in DD. The effect of *ValenceDiff* was significant ($b = 0.87$, 95% CI [0.79, 0.96], *OR=*2.39, $z = 20.35$, $p < .001$), as was the effect of *Frame* ($b = 0.77$, 95% CI [0.58, 0.95], *OR=*2.39, $z = 8.22$, $p < .001$); the interaction was not significant ($p = .120$; see the top panel of Fig. 1). Across cells, the logits of the choice proportions were correlated .95 and .92 with those from DD’s online and student samples, respectively.

### Robustness checks

The effects of *ValenceDiff* and *Frame* were significant in all four domains, all four domain positions (first to fourth), and both option orders (certain or risky first; all $\mathit{p}\mathrm{s} < .001$; the option-order analyses were not preregistered), as well as in three-level models with participants nested within microstudies (DeKay et al., 2022) or conditions (all $\mathit{p}\mathrm{s} < .001$; these models were not preregistered). See the OSM for details.

### Framing effects in matched and mismatched comparisons

The bottom panel of Fig. 1 shows that framing effects were significant in seven of the nine matched comparisons.

As DD noted, framing effects can be pushed around in mismatched pairings of gain and loss cells. For example, in the replication, the framing effect in L3–G7 was amplified ($b = 4.84$), whereas that in L7–G3 was reversed ($b = -2.96$). Both results are consistent with interval-scaled EVA, but they are based on rearrangements of information already presented in Fig. 1 (see Fig. S2 in the OSM for all 81 possible comparisons).

### Framing effects in choices between completely described options

**Fig. 2:**
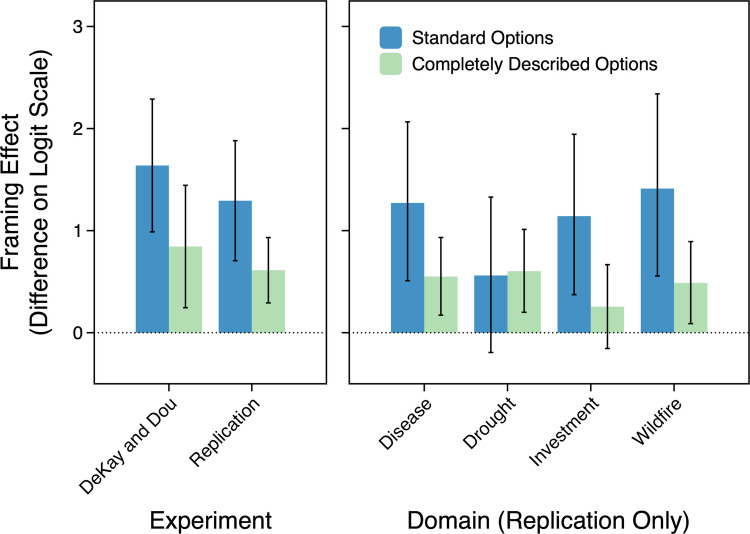
**Left:** Framing effects for the standard options (L6–G4) and completely described options (L5–G5) in DeKay and Dou’s (2024) experiment (*n*s = 153 and 97 for the two comparisons, respectively) and in the replication (*n*s = 118 and 477, respectively). DeKay and Dou’s data are collapsed across their two samples. **Right:** Framing effects for the same comparisons in each domain of the replication (*n*s = 118 and 477 in each domain). Error bars indicate 95% confidence intervals

Figure 2 shows the standard framing effect (L6–G4) and the effect for completely described options (L5–G5). The left panel highlights the similarity between DD’s results and the replication. As in DD, the framing effect in choices between complete options was significant ($b = 0.61$, 95% CI [0.29, 0.93], *OR*=1.84, $z = 3.75$, $p < .001$). This effect was significant in three of the four domains (see the right panel of Fig. 2), three of the four domain positions, and both option orders (the domain-position and option-order tests were not preregistered). In sum, DD’s intriguing result for the L5–G5 comparison replicated nicely in the much larger sample.

### Other specifications of FTT, EVA, and LBH

Mixed-effects logistic regression results for the eight specifications of FTT, EVA, and LBH appear in Table 5.^16^ The most striking feature of the table is the similarity of results across specifications: All gist, valence, and quantifier-interpretation predictors were significant at $p < .001$, and the residual framing effect was significant at $p < .001$ in all specifications except rescaled EVA.

**Table 5:** Results of mixed-effects logistic regression models for several specifications of FTT, EVA, and LBH

	Models with interactions		Models without interactions		Models without Frame	
Model and predictor	Coefficient	BIC	Coefficient	BIC	Coefficient	BIC
FTT (interpretation 1)		7695.8		7690.8		7750.3
Gist difference	0.92***		0.93***		0.92***	
Frame	0.77***		0.79***			
Interaction	0.20*					
FTT (interpretation 2)		7582.5		7579.8		*7600.4*
Gist difference	1.04***		1.05***		1.07***	
Frame	0.48***		0.50***			
Interaction	0.25*					
FTT (interpretation 3)^a^		7564.2		7557.3		7581.0
Gist difference	0.88***		0.88***		0.90***	
Frame	0.51***		0.52***			
Interaction	0.11					
EVA (interval-scaled)		*7562.6*		*7556.2*		7619.2
Valence difference	0.87***		0.87***		0.87***	
Frame	0.77***		0.78***			
Interaction	0.12					
EVA (original)		7639.5		7633.9		7693.2
Valence difference	1.26***		1.27***		1.26***	
Frame	0.76***		0.77***			
Interaction	0.23^†^					
EVA (free valence differences)		7587.7		7566.4		7629.2
Positive vs. mixed	0.86***		0.86***		0.87***	
Mixed vs. negative	0.57***		0.57***		0.55***	
Positive vs. negative	1.83***		1.83***		1.82***	
Frame	0.77***		0.78***			
Interaction (pos. vs. mix.)	0.47*					
Interaction (mix. vs. neg.)	−0.04					
Interaction (pos. vs. neg.)	0.19					
EVA (rescaled, with *f* = 0.61)^a^		**7542.4**		7535.9		**7527.4**
Rescaled valence difference	1.09***		1.09***		1.08***	
Frame	−0.07		−0.06			
Interaction	0.15					
LBH (separate effects)		7549.9		**7534.5**		7600.4
Interpretation score (risky)	1.13***		1.14***		1.12***	
Interpretation score (certain)	−0.60***		−0.60***		−0.61***	
Frame	0.77***		0.79***			
Interaction (risky)	0.14					
Interaction (certain)	−0.10					

BIC = Bayesian information criterion; FTT = fuzzy-trace theory; EVA = explicated valence account; LBH = lower-bounding hypothesis. Intercepts are omitted (they ranged from −0.86 to −0.83). Italic type indicates the best model (the lowest BIC) of each type (with or without interactions or *Frame*) among the five theory versions considered by DeKay and Dou (2024). Boldface type indicates the best model of each type among all eight theory versions. Underlined type indicates that the BIC was adjusted upward to account for the separate estimation of the additional parameter *f*. Results are based on models’ predictions for all choices

^a^This theory specification was not preregistered

^†^ *p* < .10. *p < .05. ***p < .001

Among DD’s five specifications, interval-scaled EVA had the lowest (best) BIC when *Frame* was included as a predictor, as in DD. When *Frame* was omitted, FTT2 performed best. Arguably, omitting *Frame* yields purer comparisons among theories that claim to account for framing effects.

The new FTT3 outperformed DD’s two FTT versions in all BIC comparisons, presumably because it predicts positive framing effects in four of the nine matched comparisons. However, it did not eliminate the residual framing effect.

Rescaled EVA requires more explanation. In the OSM, I show that the scaling factor $f$ can be estimated with a reparameterized model that uses “shifted” valences (denoted by *S* rather than *R*), omits *Frame* (because I wanted its coefficient to be zero), and omits the interaction (for simplicity). Ignoring the random intercept and the error term, the right-hand side of the model is:

3 $$b_0+b_1\mathit{SValence}_\mathrm{Risky}+b_2\mathit{SValence}_\mathrm{Certain},$$

with the shifted valences of both options given by:

4 $${SValence} = \left\{\begin{array}{ll}{Valence}-{1}\ & \mathrm{in\ gains}\\ {Valence}+{1}\ & \mathrm{in\ losses}.\end{array}\right.$$

As explained in the OSM, the estimate of $f$ is $-b_2/b_1$. This estimate is used to compute the rescaled valences using Eq. 1, and the rescaled valence difference, *RValenceDiff*, is used as a predictor in place of *ValenceDiff*. Finally, models’ BICs are adjusted upward to account for the separate estimation of $f$.

The reparameterized model yielded $f = 0.61$, implying certain-option valences of (+1, +0.39, −0.23) in gains and (+0.23, −0.39, −1) in losses. The −0.23 and +0.23 values are for negations in gains (e.g., “400 people will not be saved”) and losses (e.g., “400 people will not die”), respectively.^17^ With these valences, rescaled EVA outperformed all other EVA and FTT specifications in terms of BIC, and the residual framing effect was small and no longer significant.

Separate-effects LBH also performed well in terms of BIC, being trivially better than rescaled EVA in one comparison, but the residual framing effect remained significant. As in DD’s two samples, the coefficient for the risky option was nearly twice that for the certain option.^18^ Note that rescaled EVA also implies that risky options have more impact: The scaling factor $f = 0.61$ implies a ratio of $1/f = 1.64$.

## Discussion

One could not ask for stronger confirmation of DD’s results. The residual framing effect in their regression analyses and the framing effect in choices between completely described options generalize very well to US adults. These results expand the relevance of the risky-choice framing effect by demonstrating that it persists (to a reduced degree) in situations where it was thought to be eliminated.

Why do these results differ from those of earlier studies? One possibility involves the participants. None of the studies reviewed by Broniatowski and Reyna (2018) used online samples, and most previous studies with completely described options were conducted in other countries. Differences in scenarios and procedures may also matter. For example, Betsch and Kraus (1999) used videos of balls rolling down (possibly blocked) tracks. Finally, because the earlier studies were typically small ($n < 100$ for completely described options), the differences may reflect nothing more than sampling error.

### Insights for theory

Unlike PT and other mathematical theories, all tested versions of FTT, EVA, and LBH account for the effects of completely and incompletely described options reasonably well. Except for LBH, these specifications rely heavily on categorical representations, an idea first introduced in FTT. However, the only specification to account for the residual framing effect and the framing effect in choices between completely described options is a rescaled version of EVA that treats negations in certain options as less extreme than zero outcomes in risky options.

FTT3 benefits from a similar ordering of negations and zero outcomes (also see Reyna’s (2025) FTT extension). Unfortunately, competing categorical comparisons render the change ineffectual in many choices, including those between completely described options (see Footnote 14). In contrast, the change in rescaled EVA applies more generally.

The credibility of rescaled EVA is bolstered by its performance in backward-looking assessments: In DD’s two samples, rescaled EVA outperformed all other specifications of EVA and FTT (including FTT3) and eliminated the residual framing effect, even in out-of-sample tests using the scaling factor from the replication (see the OSM).

Separate-effects LHB and rescaled EVA both suggest that interpretations of incomplete risky options are more important than interpretations of incomplete certain options. However, LBH explanations for this result are undermined by Mandel’s (2014) finding that lower-bound interpretations are less likely for incomplete risky options. In rescaled EVA, the greater importance of risky options is a natural consequence of treating negations in certain options as less extreme.^19^

More broadly, the performance of rescaled EVA suggests that FTT, EVA, and related accounts could benefit from a shift toward more continuous, less categorical explanations. For example, the “evaluative polarity” (Wallin et al., 2016) or “valence intensity” (McElroy, 2024) of the words in option descriptions can affect the magnitude of framing effects. The relative extremity of negations and zero outcomes can be viewed as a similar intensity variation. A general account might entail more nuanced assessments of options’ positivity or negativity that incorporate numerical as well as linguistic features. Such efforts should be informed by ratings and other judgments regarding option descriptions and their aspects (Kühberger & Gradl, 2013; Mandel & Kelly, 2024; Peters & Levin, 2008; Reyna & Brust-Renck, 2020; Reyna et al., 2021; Tombu & Mandel, 2015).

### Constraints on generality

The results reported here may not generalize to other populations, such as children or adults in other countries, or to other problem types, such as those with intermediate “somewhat risky” options (Garge & DeKay, 2024). Negations in risky options (e.g., “a 2/3 probability that 600 people will not be saved”) may behave differently than negations in certain options. As always, one should not assume that results for between-participants comparisons hold for individuals.

## Conclusion

DeKay and Dou’s (2024) results replicated almost perfectly in a more representative sample of US adults. As those authors concluded, risky-choice framing effects are only partly explained by the valence or gist of the option descriptions. In addition to confirming the residual framing effect, the replication provides much stronger evidence for the surprising framing effect in choices between completely described options. A new rescaled version of EVA accounts for these results, while other theory specifications do not. More generally, attending to the linguistic details of option descriptions can make theories of risky choice more realistic, more applicable, and more accurate.

## Supplementary Information

Below is the link to the electronic supplementary material.

Supplementary file1 (PDF 1.10 MB)

## Data Availability

Data and materials are available via the Open Science Framework at https://osf.io/s5k3p.

## Footnotes

1 The quote is often attributed to John Wilmot, Second Earl of Rochester, but this may be implausible (Quote Investigator, 2015). Punctuation varies.

2 This is not the only way to match cells. One could instead match cells according to whether the descriptions of the risky options contain only zero outcomes, only nonzero outcomes, or both, and whether the descriptions of the certain options contain only affirmations (e.g., “will”), only negations (e.g., “will not”), or both. For example, cells G1 and L9 are matched in these respects, but not in the sense considered by DD because the outcomes are different in gains and losses. The presence or absence of zero outcomes is especially important in fuzzy-trace-theory explanations of framing effects.

3 Broniatowski and Reyna’s (2018) review also included G2 vs. L8 and G9 vs. L1, and Stocké (1998) included G3 vs. L7. The G9 vs. L1 comparisons in Broniatowski and Reyna were from Mandel (2001), who used different wording than that in Table 1. Specifically, the risky options used “all 600 people will not be saved” rather than “no people will be saved” in gains and “all 600 people will not die” rather than “no people will die” in losses. In accord with my replication goal, I used DD’s wording in the research reported here.

4 In risky options, the *no* in *no people* is also a negation. To differentiate the possibilities, I use the term *zero outcome* for components that include *no* (here, in risky options) and the term *negation* for components that include *not* (here, in certain options).

5 In a sophisticated comparison of theoretical explanations for a different variant of the standard framing effect, Huizenga et al. (2023) reported that most participants followed an FTT-based hybrid theory with two steps. The first step is a heuristic involving the probability of the better outcome in the risky option; it operates similarly in both frames and cannot lead to a framing effect. The second step, if used, is FTT’s categorical gist. Extending the hybrid theory to the cells of Table 1 would also require specifying how comparisons between negations and zero outcomes are handled. I do not consider this theory further.

6 Wallin et al.’s (2016) relative-evaluative-polarity account is essentially the same as EVA, with options having positive, negative, or mixed polarity. Wallin et al.’s account also allows for a weaker, direct effect of frame (gains vs. losses) on choice, but the authors did not suggest a mechanism for the effect or provide any evidence for it.

7 Fisher (2022) argued that FTT entails a version of LBH because *some* must mean *some or all* when applied to all 600 people in the risky option. Reyna (2025) acknowledged that *some* includes *all*, at least in FTT’s binary distinction between *some* and *none*. FTT holds that the categorical gist used in decision making is the simplest distinction that discriminates between options. For most framing problems, that is the binary distinction rather than the trinary distinction between *all*, *some*, and *none*. Broniatowski and Reyna (2018, p. 242) noted that the trinary distinction mimics FTT’s ordinal comparisons, which rarely if ever predict framing effects.

8 Once adopted, interpretations of certain and risky options had similar effects on choice (Mandel, 2014).

9 Simultaneously, I collected data from 277 additional participants in two conditions of a different experiment (preregistered at https://aspredicted.org/hym6-cnd4.pdf). The target sample size for both experiments was 1,920 after exclusions or 2,133 if a 10% exclusion rate is assumed. Connect’s maximum of 2,214 for census-matched samples was only slightly higher, so I used that as the target. The total initial sample size was 2,220. The demographics for the full sample were slightly closer to the census targets (e.g., perfect for gender and race). See the Online Supplemental Material (OSM) for details.

10 Kumle et al.’s (2021) procedures require a slope *b* rather than a standardized effect size like *d*. Following DD, I used a simple conversion appropriate for logistic regression, $b = d\times \pi /\sqrt{3}$ (Borenstein et al., 2009). However, this conversion does not account for the variance of the random intercept. Doing so yields slightly larger slopes and thus slightly higher power (e.g., .89 instead of .84 for *d* = 0.2 and *n* = 480). See the OSM for details.

11 A larger estimate for the intercept variance also lowered these power estimates. Accounting for that variance in the conversion from *d* to *b* increased the power estimates to .90 for *d* = 0.5 and .27 for *d* = 0.2. See the OSM.

12 A reviewer suggested that *none saved* in the risky option should really be *some probability of none saved*, in which case the ordering in FTT3 is not so intuitive (but note that if the comparison is indeterminate, FTT2 results). However, FTT does not typically distinguish between certain and probabilistic outcomes in categorical comparisons; certain is just *some chance* (Broniatowski & Reyna, 2018). Regardless, the ordering in FTT3 is worth considering as an empirical possibility. Coincidentally, Reyna’s (2025) recent extension of FTT uses the same ordering as FTT3 (see her Table 2), with Aristotle’s square of opposition providing the rationale.

13 A similar version without amplification (called FTT4 in the OSM) performed poorly.

14 Consider the gain frame (G5). With subscripts to facilitate comparisons, the gist of the certain option is *some saved*_1_* and some not saved*_2_, and the gist of the risky option is *some saved*_3_* or none saved*_4_. The comparison between *some saved*_1_ and *some saved*_3_ favors neither option (predicted preference = 0), the comparison between *some saved*_1_ and *none saved*_4_ favors the certain option (‒1), and the comparison between *some not saved*_2_ and *some saved*_3_ favors the risky option (+1). There is already a contradiction (‒1 vs. +1), so the overall comparison is indeterminate (0) regardless of how the comparison between *some not saved*_2_ and *none saved*_4_ is resolved.

15 I mean prediction in the statistical sense, as opposed to foresight. I considered this EVA specification (along with FTT3) only after the data were collected.

16 Results from mixed-effects linear models for predicting participants’ strength-of-preference ratings were very similar (see Table S16 in the OSM). For example, orderings of theory versions by their BICs were nearly identical to those in the columns of Table 5, with the only reversal involving the virtual tie between FTT2 and separate-effects LBH in the rightmost column of Table 5.

17 The zero point need not be meaningful in interval-scaled or rescaled EVA, so these valences do not imply that (for example) “400 people will not be saved” is 23% as bad as “a 2/3 probability that no people will be saved.” A better interpretation, consistent with interval scaling, is that the difference between “200 people will be saved” and “400 people will not be saved” is about 61% as large as the difference between “200 people will be saved” and “a 2/3 probability that no people will be saved.”

18 I also preregistered an LBH version in which the ratio of the risky- and certain-option coefficients was fixed to 2. The reduction in parameters yielded the lowest BIC among models with *Frame* but not among models without *Frame* (BICs = 7532.5, 7529.9, and 7591.9 for the three columns of Table 5, respectively). This was the only theory version that relied on DD’s data for a parameter estimate or constraint. Aside from the remarkable similarity of the results to DD’s, the take-home message is not clear.

19 For the standard framing effect, Kühberger and Gradl (2013) found that interpretations of the certain option are more important than those of the risky option. There is no contradiction. In the standard effect (L6–G4), the valence (or lower-bound interpretation) of the incompletely described certain option varies greatly across frames, while that of the completely described risky option does not.

## Code availability

Analysis scripts are available via the Open Science Framework at https://osf.io/s5k3p.
