# Supplementary material for: Risky-choice framing effects persist when option descriptions are matched and complete: A replication and extension of DeKay and Dou (2024)
Source: Psychon Bull Rev. 2026 Apr 14;33(4):139. doi: 10.3758/s13423-025-02771-w (PMC13079499; doi:10.3758/s13423-025-02771-w)
Supplement: Supplementary file 1 — Supplementary file1 (PDF 1.10 MB) [file 13423_2025_2771_MOESM1_ESM.pdf]

Supplemental Material for

"Risky-Choice Framing Effects Persist When Option Descriptions are Matched and Complete: A Replication and Extension of DeKay and Dou (2024)"

Michael L. DeKay
Department of Psychology, The Ohio State University

The sections in this file present topics in the roughly same order as in the main text:

Deviations From the Preregistration
Predictions of Different Specifications of Fuzzy-Trace Theory, the Explicated Valence Account, and the Lower-Bounding Hypothesis for Variants of the Original Disease Problem
Matching the Sample to U.S. Census Data
  U.S. Census Estimates
  Sampling on Connect
Participant Exclusions
Example Scenarios and Options in the Four Domains
Power Analyses
  Converting a Standardized Effect-Size Measure to an Unstandardized Slope
  Power for Choices With Completely Described Options
  Power for Other Matched Comparisons
  Power for the Standard Framing Effect
  Power for the Primary Analysis Using the Full Design
Robustness Checks for the Primary Interval-Scaled EVA Model
  Separate Analyses for Each Domain, Domain Position, and Option Order
  Three-Level Models With Participants Nested Within Microstudies or Conditions
Framing Effects in All 81 Possible Cell Pairs
Robustness Checks for Framing Effects in Choices Between Completely Described Options
Additional Analyses for FTT and EVA, Assuming "Some Not Saved" > "None Saved" and "None Die" > "Some Do Not Die"
  Fuzzy-Trace Theory
  The Explicated Valnce Account
  Performance of the New FTT and EVA Models in DeKay and Dou's (2024) Samples
Lower-Bounding Specifications With Different Weights for Interpretations of Certain and Risky Options
Mixed-Effects Linear Regressions for Predicting Participants' Strength-of-Preference Ratings
Additional References Not Cited in the Main Text

During the review and revision process, substantial material from the Supplemental Material was incorporated into the main text. I have not tried to eliminate all redundancies. In some cases, the Supplemental Material may still include more detail or context.

Deviations From the Preregistration

Table S1 lists the deviations from the preregistration materials. Almost all deviations involved additional analyses intended to assess the robustness of the main conclusions or test new models that might explain the residual framing effect.

Table S1

Deviations from Preregistration Materials

Deviation type	Description
Robustness check	I tested whether domain, domain position, and option order moderated the effects of *ValenceDiff* and *Frame*.
Robustness check	I tested whether the effects of *ValenceDiff* and *Frame* were significant in each of the two option orders (see Table S7).
Robustness check	I ran three-level mixed-effects models with participants nested within microstudies or conditions (see Table S8).
Reality check	I tested whether the framing effect was significantly smaller in choices with completely described options (L5–G5) than in choices with the standard options (L6–G4).
Robustness check	I tested whether domain, domain position, and option order moderated the framing effect in choices with completely described options.
Robustness check	I tested the framing effect in choices with completely described options separately in each of the four domains, each of the four domain positions, and each of the two option orders (see Tables S9, S10, and S11).
Robustness check	I compared the predictive ability of various specifications of FTT, EVA, and LBH in models without the *Frame* predictor (see the rightmost columns of Table 5 in the main text).
New explanatory models	I tested new versions of FTT and EVA in which "some not saved" is preferred to "none saved" and "none die" is preferred to "some do not die" (see Table S12).
New explanatory models	I tested the new versions of FTT and EVA in which "some not saved" is preferred to "none saved" and "none die" is preferred to "some do not die" in DeKay and Dou's (2024) two samples (see Tables S13 and S14).
Robustness check	I ran mixed-effects linear regression models using participants' strength-of-preference ratings as the dependent variable (see Table S16).

Note. FTT = fuzzy-trace theory. EVA = explicated valence account. LBH = lower-bounding hypothesis.

Predictions of Different Specifications of Fuzzy-Trace Theory, the Explicated Valence Account, and the Lower-Bounding Hypothesis for Variants of the Original Disease Problem

Table 2 in the main text provides option descriptions and predicted preferences from different specifications of fuzzy-trace theory (FTT), the explicated valence account (EVA), and the lower-bounding hypothesis (LBH) for all 18 cells of the design. This section provides additional detail.

Fuzzy-Trace Theory

DeKay and Dou (2024) used two versions of FTT that differ depending on how one interprets the categorical comparison between (for example) “no people will be saved” and “some people will not be saved” in gains and between “no people will die” and “some people will not die” in losses. Broniatowski and Reyna (2018) did not address this comparison directly, perhaps because it was not necessary for FTT predictions in the cases they considered. However, they did state that (a) “no people will be saved” is a subset of “some people will be saved” and (b) “some people will be saved” is categorically preferred to “some people will not be saved.” By the usual logic of set theory, it follows that (c) “no people will be saved” is categorically preferred to “some people are not saved,” even though that sounds counterintuitive. Applying the same logic in losses implies that “some people will not die” is categorically preferred to “no people will die,” even though that sounds counterintuitive. This version of FTT is called FTT1. In the alternative version, FTT2, the categories in question are equally preferred (or perhaps not comparable), in which case such comparisons play no role in determining categorical preferences between options. DeKay and Dou provided a comprehensive discussion of the predictions of

both versions of FTT for the 18 cells of the design in about 12 pages of their Supplemental Material. Readers are referred to that document for details.

I created two new versions of FTT, which I call FTT3 and FTT4 to distinguish them from DeKay and Dou's (2024) FTT1 and FTT2. The better of the two new versions, FTT3, is the one highlighted in the main text.

FTT3 and FTT4 both differ from DeKay and Dou's (2024) FTT1 and FTT2 in the way they handle negations. I start with FTT4, because it is a bit simpler than FTT3. Following Kühberger and Tanner's (2010) intuition, I treat "some people will not be saved" as categorically better than "no people will be saved," and I treat "some people will not die" as categorically worse than "no people will die." In gains, there are three categories of outcomes, with the preference order "some will be saved" > "some will not be saved" > "none will be saved." In losses, there are three categories of outcomes, with the preference order "none die" > "some will not die" > "some will die." In other scenarios, the word "lost" is used in the loss frame.

Unlike Broniatowski and Reyna (2018), I do not treat any category as being a subset of any other. As a result, interval comparisons are usually not allowed because the relevant outcomes are in disjoint categories. Only the categorical comparisons matter. In each choice, all possible categorical comparisons between outcomes are coded +1 if they favor the risky option, –1 if they favor the certain option, and 0 if they favor neither option (see Table S2). As in Broniatowski and Reyna, a score of 0 does not conflict with scores of +1 or –1, but +1 and –1 conflict with each other. If there is any conflict, the comparison is indeterminant. The resulting preference predictions are shown in the column for FTT4 in Table S2. For matched comparisons, framing effects are predicted in L1–G1, L4–G4, L6–G6, and L9–G9. These framing effects are predicted

to be half as large as that for the standard comparison (L6–G4). No framing effect is predicted in the other six matched comparisons.

Table S2

Option Descriptions and Predicted Preferences From Fuzzy-Trace Theory for Each Cell for a Risky-Choice Framing Problem with 600 Lives at Risk, Assuming "Some Not Saved" > "None Saved" and "None Die" > "Some Do Not Die"

			Predicted preference for the risky option		
Cell	Certain option	Risky option	Categorical comparisons	FTT3	FTT4
		Gains			
G1	200 people will be saved	a 1/3 probability that 600 people will be saved	0	0	0
G2	200 people will be saved and 400 people will not be saved	a 1/3 probability that 600 people will be saved	0, +1	+1	+1
G3	400 people will not be saved	a 1/3 probability that 600 people will be saved	+1	+2	+1
G4	200 people will be saved	a 1/3 probability that 600 people will be saved and a 2/3 probability that no people will be saved	0, –1	–1	–1
G5	200 people will be saved and 400 people will not be saved	a 1/3 probability that 600 people will be saved and a 2/3 probability that no people will be saved	0, –1, +1, **–1**	0	0
G6	400 people will not be saved	a 1/3 probability that 600 people will be saved and a 2/3 probability that no people will be saved	+1, **–1**	0	0
G7	200 people will be saved	a 2/3 probability that no people will be saved	–1	–2	–1
G8	200 people will be saved and 400 people will not be saved	a 2/3 probability that no people will be saved	–1, **–1**	–1	–1
G9	400 people will not be saved	a 2/3 probability that no people will be saved	**–1**	–1	–1
		Losses			
L1	200 people will not die	a 1/3 probability that no people will die	**+1**	+1	+1
L2	200 people will not die and 400 people will die	a 1/3 probability that no people will die	**+1**, +1	+1	+1
L3	400 people will die	a 1/3 probability that no people will die	+1	+2	+1
L4	200 people will not die	a 1/3 probability that no people will die and a 2/3 probability that 600 people will die	**+1**, –1	0	0
L5	200 people will not die and 400 people will die	a 1/3 probability that no people will die and a 2/3 probability that 600 people will die	**+1**, –1, +1, 0	0	0
L6	400 people will die	a 1/3 probability that no people will die and a 2/3 probability that 600 people will die	+1, 0	+1	+1
L7	200 people will not die	a 2/3 probability that 600 people will die	–1	–2	–1
L8	200 people will not die and 400 people will die	a 2/3 probability that 600 people will die	–1, 0	–1	–1
L9	400 people will die	a 2/3 probability that 600 people will die	0	0	0

Note. FTT3 and FTT4 = two new interpretations of fuzzy-trace theory. Higher predicted preference values indicate stronger preferences for the risky option. Boldface type indicates categorical comparisons between "not" and "none."

FTT3 is like FTT4, but it allows for stronger preferences when the absence of the nonzero outcome in the risky option highlights the some-versus-none comparison in cells G7 and L3, as in several previous articles on FTT (Chick et al., 2016; Reyna, 2012; Reyna & Brainerd, 1995; Reyna et al., 2014, 2021). In FTT3, I assume that the same highlighting occurs for the some-versus-not comparisons in cells G3 and L7 (see Table S2). Because these changes affect matched cells in gains and losses, the predicted framing effects in matched comparisons are the same as for FTT4. (Highlighting some-versus-none comparisons but not some-versus-not comparisons would lead to predicted framing effects in L3–G3 and L7–G7 as well, but I cannot think of a principled reason for making such a distinction.)

The Explicated Valence Account

Interval-scaled EVA is described in some detail in the main text (see the scores in Table 1). Original EVA treats all categorical differences similarly, with no differences in magnitude, so differences between positive and negative valences are coded ±1 rather than ±2. A third "free valence differences" version of EVA uses different codes for positive-versus-mixed, mixed-versus-negative, and positive-versus-negative comparisons (see Table 2 in the main text). On each code, cells are coded +1 if the difference favors the risky option, –1 if it favors the certain option, and zero otherwise. On the first code (positive vs. mixed), cells G2 and L2 are coded +1, and cells G4 and L4 are coded –1. On the second code (mixed vs. negative), cells G6 and L6 are coded +1, and cells G8 and L8 are coded –1. On the third code (positive vs. negative), cells G3 and L3 are coded +1, and cells G7 and L7 are coded –1. For all three codes, cells with valence differences of zero are among those assigned values of zero, so the coefficient of each code gives the effect of a 1-unit valence difference of the specified type, relative to the matched-valence cells.

In a new version of EVA, which I call *rescaled* EVA, I code the negations in certain options as less extreme, in accord with Kühberger and Tanner's (2010) intuition. For example, "400 people will not be saved" might be coded –1/3 rather than –1, and "200 people will not die" might be coded +1/3 rather than +1. As before, I code the completely described options as the average of the codes for the two aspects. In gains, the original valences (+1, 0, –1) would become (+1, +1/3, –1/3). In losses, the original valences (+1, 0, –1) would become (+1/3, –1/3, –1). For the risky options, the codes remain (+1, 0 –1) in both gains and losses.

More generally, the valences for the certain option are rescaled linearly, but differently in gains and losses. In gains,

$$RValence_{\text{Certain}} = f \times Valence_{\text{Certain}} + (1 - f). \qquad \text{(S1)}$$

The *R* indicates the rescaled valence, with *f* as the scaling factor. In the above example, f = 2/3. In losses,

$$RValence_{\text{Certain}} = f \times Valence_{\text{Certain}} - (1 - f). \qquad \text{(S2)}$$

For the risky option, the valences remain the same as before, in both frames:

$$RValence_{\text{Risky}} = Valence_{\text{Risky}}. \qquad \text{(S3)}$$

The difference between the rescaled valences is a rescaled valence difference variable:

$$RValenceDiff = RValence_{\text{Risky}} - RValence_{\text{Certain}}. \qquad \text{(S4)}$$

The results of Equations S1–S4 for each of the 18 cells are shown in Table S3. From Equations S1, S2, and S4 (or from the values in the rightmost column of Table S3), it is easy to calculate the predictor of the framing effect, FE_{Pred}, as in the main text. With the rescaled valences, and skipping some steps, FE_{Pred}, is the same for every matched comparison (e.g., L1–G1, L2–G2):

$$FE_{\text{Pred}} = \frac{RValenceDiff_{\text{Losses}} - RValenceDiff_{\text{Gains}}}{2} = \frac{2(1 - f)}{2} = 1 - f. \qquad \text{(S5)}$$

Table S3

Option Descriptions and Predicted Preferences From Rescaled EVA for Each Cell for a Risky-Choice Framing Problem with 600 Lives at Risk, Assuming "Some Not Saved" > "None Saved" and "None Die" > "Some Do Not Die"

			Rescaled valences and valence difference in favor of the risky option		
Cell	Certain option	Risky option	Certain	Risky	Risky – Certain
		Gains			
G1	200 people will be saved	a 1/3 probability that 600 people will be saved	$+1$	$+1$	0
G2	200 people will be saved and 400 people will not be saved	a 1/3 probability that 600 people will be saved	$1-f$	$+1$	f
G3	400 people will not be saved	a 1/3 probability that 600 people will be saved	$1-2f$	$+1$	$2f$
G4	200 people will be saved	a 1/3 probability that 600 people will be saved and a 2/3 probability that no people will be saved	$+1$	0	-1
G5	200 people will be saved and 400 people will not be saved	a 1/3 probability that 600 people will be saved and a 2/3 probability that no people will be saved	$1-f$	0	$-1+f$
G6	400 people will not be saved	a 1/3 probability that 600 people will be saved and a 2/3 probability that no people will be saved	$1-2f$	0	$-1+2f$
G7	200 people will be saved	a 2/3 probability that no people will be saved	$+1$	-1	-2
G8	200 people will be saved and 400 people will not be saved	a 2/3 probability that no people will be saved	$1-f$	-1	$-2+f$
G9	400 people will not be saved	a 2/3 probability that no people will be saved	$1-2f$	-1	$-2+2f$
		Losses			
L1	200 people will not die	a 1/3 probability that no people will die	$-1+2f$	$+1$	$2-2f$
L2	200 people will not die and 400 people will die	a 1/3 probability that no people will die	$-1+f$	$+1$	$2-f$
L3	400 people will die	a 1/3 probability that no people will die	-1	$+1$	2
L4	200 people will not die	a 1/3 probability that no people will die and a 2/3 probability that 600 people will die	$-1+2f$	0	$1-2f$
L5	200 people will not die and 400 people will die	a 1/3 probability that no people will die and a 2/3 probability that 600 people will die	$-1+f$	0	$1-f$
L6	400 people will die	a 1/3 probability that no people will die and a 2/3 probability that 600 people will die	-1	0	1
L7	200 people will not die	a 2/3 probability that 600 people will die	$-1+2f$	-1	$-2f$
L8	200 people will not die and 400 people will die	a 2/3 probability that 600 people will die	$-1+f$	-1	$-f$
L9	400 people will die	a 2/3 probability that 600 people will die	-1	-1	0

Note. EVA = explicated valence account. f = scaling factor. Higher valence differences indicate stronger preferences for the risky option.

With $f = 2/3$, as in the above example, the predicted framing effect in matched comparisons (and thus for the entire experiment) is 1/3 as large as that the standard framing effect (L6–G4). This result in Equation S5 suggests that the proposed rescaling could account for the framing effect in DeKay and Dou's (2024) experiment and the replication.

The goal is to find the value of *f* that makes the residual framing effect disappear. Consider a stripped-down version of the right-hand side of a mixed-effects logistic regression equation, with the random intercept and error term omitted. I omit the *Frame* predictor because I want its coefficient to be zero. For simplicity, I also omit the *RValenceDiff* × *Frame* interaction.

$$b_0 + b_1 RValenceDiff \tag{S6}$$

$$b_0 + b_1\left(RValence_{\text{Risky}} - RValence_{\text{Certain}}\right) \tag{S7}$$

In gains, substituting the expressions in Equations S1 and S3 yields:

$$b_0 + b_1\left[Valence_{\text{Risky}} - (f \times Valence_{\text{Certain}} - f + 1)\right] \text{ or} \tag{S8}$$

$$b_0 + b_1\left(Valence_{\text{Risky}} - 1\right) - fb_1(Valence_{\text{Certain}} - 1). \tag{S9}$$

In losses, substituting the expressions in Equations S2 and S3 yields:

$$b_0 + b_1\left[Valence_{\text{Risky}} - (f \times Valence_{\text{Certain}} + f - 1)\right] \text{ or} \tag{S10}$$

$$b_0 + b_1\left(Valence_{\text{Risky}} + 1\right) - fb_1(Valence_{\text{Certain}} + 1). \tag{S11}$$

Equations S9 and S11 suggest that new versions of the valence variables would be useful.

$$SValence_{\text{Certain}} = Valence_{\text{Certain}} \pm 1 \tag{S12}$$

$$SValence_{\text{Risky}} = Valence_{\text{Risky}} \pm 1 \tag{S13}$$

In the above expressions, *S* stands for “shifted,” with -1 used in gains, and $+1$ used losses. Equations S9 and S11 can now be expressed in terms of the shifted valences:

$$b_0 + b_1\left(SValence_{\text{Risky}}\right) - fb_1(SValence_{\text{Certain}}). \tag{S14}$$

Equation S14 can be rewritten with separate coefficients for the risky and certain terms:

$$b_0 + b_1\left(SValence_{\text{Risky}}\right) + b_2(SValence_{\text{Certain}}). \tag{S15}$$

Equation S15 is a reparameterization of Equation S6 or S7. Because $b_2 = -fb_1$ (compare Equations S14 and S15), fitting Equation S15 to the data yields the desired estimate of *f*:

$$f = -\frac{b_2}{b_1}. \quad \text{(S16)}$$

This value of f can be used to compute the rescaled certain-option valences differences using Equations S1 and S2, and the rescaled valence difference from Equation S4 can then be used as a predictor in regressions to predict participants' choices. The BICs for those regressions should be increased to account for the separate estimation of the additional parameter f.

The Lower-Bounding Hypothesis

This section presents additional preregistered theory versions involving different weights for the valences (or interpretations) of certain and risky options. Because the motivation may not be obvious, an explanation is warranted.

Numerous authors have suggested that incompletely described options are ambiguous (Fisher & Mandel, 2021; Kühberger & Tanner, 2010; Mandel, 2001, 2014; Okder, 2012; Stocké, 1998). In the original disease problem, for example, "200 people will be saved" might be interpreted as "*at least* 200 people will be saved" for logical reasons (e.g., some of the other 400 might be saved as well) or closely related linguistic reasons (see Mandel, 2014, for a detailed discussion). Similarly, "400 people will die" might be interpreted as "*at least* 400 people will die." If there are no analogous effects for the traditional risky options, which are completely described, the apparent differences in the expected values of the certain and risky options are sufficient to explain the classic framing effect. This account is called the lower-bounding hypothesis (LBH; Fisher, 2022; Fisher & Mandel, 2021; Mandel & Kelly, 2024).

Other researchers (Chick et al., 2016; Reyna et al., 2021) have demonstrated that framing effects persist when participants are trained to correctly complete any incomplete option descriptions, implying that ambiguity is not a necessary condition for framing effects. However, I did not (and DeKay and Dou, 2024, did not) provide such training, so the incompletely

described options remained ambiguous in these experiments, implying that lower-bound interpretations were possible. Interestingly, the “at least” interpretation implies that the coding of certain options should be the same as the coding used in Table 1 of the main text: positive for good aspects, negative for bad aspects, and (presumably) zero for completely described options with both aspects.

Similar logic applies to incomplete descriptions of risky options, in two ways. First, one can apply the “at least” reasoning to the probability in an incompletely described option, so that (for example) “a 1/3 probability that 600 people will be saved” might be interpreted as “*at least* a 1/3 probability that 600 people will be saved,” which is clearly better. Similarly, “a 2/3 probability that 600 people will die” might be interpreted as “*at least* a 2/3 probability that 600 people will die,” which is clearly worse. The implication is that the coding of risky options should be like that in Table 1 of the main text: positive for good aspects, negative for bad aspects, and (presumably) zero for completely described options with both aspects. However, based on earlier results (Mandel, 2014, Experiment 3, especially Table 4), it seems likely that fewer people would apply the “at least” reasoning to incomplete risky options than to incomplete certain options.

A different line of reasoning leads to a similar coding. Kühberger and Tanner (2010) noted that an incompletely described risky option, such as “a 1/3 probability that 600 people will be saved,” does not specify anything about what happens with the remaining probability. There could be a 2/3 probability that no people will be saved, but there could instead be a 2/3 probability that some other number of people (e.g., 1, 10, 100) will be saved. There could even be a distribution, with the 2/3 probability distributed over many possible outcomes. In Kühberger and Tanner’s words, “Any number of lives, at any probability level, is possible” (p. 320). Any of these interpretations would increase the apparent expected value of options in which only the

good aspect is specified and, similarly, decrease the apparent expected value of options in which only the bad aspect is specified. The coding would again be like that in Table 1 of the main text: positive for good aspects, negative for bad aspects, and (presumably) zero for completely described options with both aspects.

I agree with Kühberger and Tanner (2010) that ambiguity is probably more of a problem for incompletely described risky options than for incompletely described certain options. However, setting this intuition aside for the moment, assume that ambiguity has equal effects for certain and risky options. If so, the ambiguity-based coding of the options would be identical to that in Table 1 of the main text. If predicted preferences are computed in the same way as in that table, as the score for the risky option minus the score for the certain option, the implications for choice proportions in the 18 cells of the design are identical to those from interval-scaled EVA. Simpler comparisons that replace differences of ±2 with differences of ±1 yield predictions identical to those from original EVA. In other words, extending the ambiguity-based lower-bounding arguments of Mandel (2014) and others to the full experimental design leads to the same predictions as DeKay and Dou's (2024) versions of Tombu and Mandel's (2015) EVA. This is worth pointing out because Tombu and Mandel did not mention Mandel's (2014) lower-bound reasoning in their development of EVA.

The foregoing arguments suggest that the effect of *ValenceDiff* in DeKay and Dou's (2024) experiment and in the current replication (see the top panel of Figure 1 in the main text) could be due to categorical comparisons, lower bounding, or some combination of the two. I suspect that the effect is not entirely due to lower bounding. According to the LBH explanation for the framing effect in the original disease problem, specifying that *exactly* 200 people will be saved in the gain frame and that *exactly* 400 people will die in the loss frame should eliminate any

ambiguity and thus eliminate the framing effect. Although Mandel (2014) reported a nonsignificant framing effect (a 16 percentage-point difference between the two frames) with such wording, Simmons and Nelson (2013) reported a significant framing effect (a 34 percentage-point difference; see Mandel, 2013/2020, for further discussion). Moreover, as DeKay and Dou noted, neither categorical comparisons nor ambiguity can account for the residual framing effect in the 18-cell experiment because the balanced design leads to identical predictions in gains and losses (i.e., the ambiguity is not asymmetric). Similarly, neither categorical comparisons nor ambiguity can account for the observed framing effect when both options are completely described, as in cells G5 and L5.

That is not to say there is no role for ambiguity or lower bounding, especially in the interpretation of incompletely described risky options. If ambiguity is a bigger problem for incompletely described risky options than for incompletely described certain options (Kühberger & Tanner, 2010), the valence scores for risky options in Table 1 of the main text should have a larger effect on choices than the valence scores for certain options do. However, the *ValenceDiff* variable of the interval-scaled EVA approach, which equals $Valence_{\text{Risky}} - Valence_{\text{Certain}}$, implicitly assumes equal and opposite effects.

I took two approaches to assessing whether the valences of risky options mattered more than the valences of certain options (or, in the language of LBH, whether interpretations of risky options mattered more than those of certain options). Both approaches were preregistered. In the first approach, I used the interpretation scores for risky options and certain options ($Interpretation_{\text{Risky}}$ and $Interpretation_{\text{Certain}}$, or equivalently, $Valence_{\text{Risky}}$ and $Valence_{\text{Certain}}$) as separate predictors. In the main text, I call this model "separate-effects LBH." My expectations were that the coefficient for risky options would be positive, the coefficient for certain options

would be negative, and that the former would be substantially larger than the latter. To assess the robustness of the results, I used three specifications of the model. The third model, which omitted the *Frame* predictor, was not preregistered.

In the second approach, I constrained the magnitude of the coefficient for risky options to be exactly twice that of the coefficient for certain options. To do so, I created a new variable, $InterpretationDiff_{2x} = 2 \times Interpretation_{Risky} - Interpretation_{Certain}$. If the coefficient of this variable is b, it follows that $b \times InterpretationDiff_{2x} = b \times (2 \times Interpretation_{Risky} - Interpretation_{Certain}) = 2b \times Interpretation_{Risky} - b \times Interpretation_{Certain}$. Once the value of b is obtained from the regression, the coefficient of $Interpretation_{Risky}$ is $2b$ and the coefficient of $Interpretation_{Certain}$ is $-b$. I call this specification "constrained-effects LBH." Note that separate-effects LBH and constrained-effects LBH can also be considered versions of interval-scaled EVA because they still treat positive-versus-negative valence differences (or interpretation differences) as being twice as large as positive-versus-mixed and mixed-versus-negative differences. Also, note that rescaled EVA also predicts a larger role for risky options than for certain options when the scaleing factor f in Equations S1 and S2 is less than 1.

Based on reanalysis of DeKay and Dou's (2024) data, I expected these models to fit better in terms of BIC than the corresponding interval-scaled EVA models (specifically, the primary model in the main text), and that the version using $InterpretationDiff_{2x}$ might outperform the version with separate coefficients for $Interpretation_{Risky}$ and $Interpretation_{Certain}$ (see the preregistration). However, all the LBH models still predict no framing effects in matched comparisons (e.g., L1–G1).

Matching the Sample to U.S. Census Data

An attractive feature of CloudResearch's Connect platform is that it offers the option of a "census matched template" for matching a sample to U.S. Census data on age (four categories), gender (two categories), race (three categories), and ethnicity (Hispanic or not) at no cost. There are also many other targeting options (e.g., state of residence, income, education).

However, at least at the time of the replication, Connect's default percentages did not match those for the U.S. adult population (see Table S4). For example, Connect's default for gender was an exact 50:50 mix of men and women. Their defaults would also underrepresent older Americans. I chose not to use Connect's defaults. Instead, I computed target percentages based on the most recent U.S. Census data I could find (details below). In addition (because it was free), I attempted to match U.S. Census data for three other variables: geographic region (four categories), personal income (three categories), and education (three categories).

Unfortunately, my enthusiasm for matching the U.S. Census on seven variables ran headlong into the limitations of Connect's participant pool, in large part because my intended sample size was pegged to the largest sample that Connect would allow for census matching (2,214 at the time). As a result, I had to abandon some targets in the latter stages of the survey (details below). Even so, the resulting percentages in the sample were close or very close to the targets for all variables except age (there were too few older participants) and education (there were too few participants with a high-school diploma or less and too many participants with a college degree or higher; see Table S4).

Table S4

Demographic Categories, Target Percentages, and Obtained Percentages

Variable and category	U.S. Census estimates for ages 18–99	Connect's "census-matched" defaults	Full sample before exclusions (*N* = 2,220)	Study sample before exclusions (*N* = 1,943)	Study sample after exclusions (*N* = 1,697)
Age					
18–29	20.1%	**22%**	22.1%	22.2%	20.3%
30–44	25.9%	**26%**	27.2%	27.4%	28.0%
45–59	23.3%	**26%**	26.3%	25.7%	26.5%
60–99	30.7%	**26%**	24.4%	24.6%	25.2%
Mean	48.7		44.9	44.9	45.4
Median	47.1		45	45	45
Range			18–99	18–99	18–86
Gender					
Female	**51.0%**	50%	51.0%	50.7%	52.0%
Male	**49.0%**	50%	49.0%	49.3%	48.0%
Race					
Black or African Am. (alone)	**13.1%**	14%	13.1%	13.4%	12.2%
White (alone)	**76.9%**	78%	76.9%	76.7%	77.4%
Other (including mixed race)	**10.0%**	8%	10.0%	9.9%	10.4%
Ethnicity					
Hispanic	**17.2%**	16%	15.2%	15.2%	15.0%
Not Hispanic	**82.8%**	84%	84.8%	84.8%	85.0%
Census region					
Midwest	**20.5%**		20.3%	20.3%	20.7%
Northeast	**17.4%**		18.1%	18.5%	18.2%
South	**38.6%**		39.7%	39.4%	39.1%
West	**23.6%**		21.9%	21.8%	22.0%
Personal income					
Less than $20,000	**31.3%**		29.5%	29.3%	29.7%
$20,000 to $59,999	**38.0%**		40.6%	41.0%	41.4%
$60,000 or more	**30.7%**		29.9%	29.6%	28.9%
Education					
High-school diploma or less	**38.8%**		19.9%	19.9%	20.4%
Assoc. degree or some college	**26.4%**		32.0%	31.9%	32.2%
College degree or higher	**34.8%**		48.1%	48.2%	47.3%

Note. Boldface type indicates the initial target percentages for the survey. I used Connect's default percentages for age because any attempt to increase the percentage in the highest age category led to a message that the survey was not feasible. Am. = American.

U.S. Census Estimates

Age and Gender

Data for age and gender were from the U.S. Census Bureau (2024, November 25). The page appears to have been updated since I retrieved the data in May, 2024. I used a table titled "Annual Estimates of the Resident Population by Single Year of Age and Sex for the United

States: April 1, 2020 to July 1, 2023 (NC-EST2023-AGESEX-RES)," which should still be the same. For age, I summed the 2023 counts (the most recent) in each of Connect's four age categories and divided by the total. For gender, I summed the 2023 counts for ages 18–99 for men and women and divided by the total. I excluded ages ≥ 100 because Connect's highest category is capped at 99. Note that the U.S. Census includes only two categories for gender. I reluctantly excluded other genders on Connect. Finally, I used the unrounded proportions to target a specific number of participants (out of 2,214) in each category (e.g., 1,129 women and 1,085 men). I used similar procedures to determine participant targets for the other demographic variables below. When creating categories in Connect, I always excluded "Prefer not to say."

Unfortunately, I was not able to use the calculated percentages and corresponding participant targets for age. Any attempt to increase the number of participants in the highest age category (60–99) resulted in a message that the survey was not feasible. I assume this difficulty was related to my using the maximum number of participants and that I could have targeted a higher percentage of older adults if my overall sample had been smaller. Not wishing to decrease the sample size, I used Connect's default target percentages even though they differed from the census data by 4.7 percentage points for the highest age category (see Table S4). Sigh.

Race and Ethnicity

Data for race and ethnicity were also from the U.S. Census Bureau (2024, November 25). I used a table titled "Annual Estimates of the Resident Population by Sex, Age, Race, and Hispanic Origin for the United States: April 1, 2020 to July 1, 2023 (NC-EST2023-ASR6H)." For race, I used the 2022 counts (the most recent) for ages 18 and over from the tabs for each race. To match Connect's default "Other" category, I summed the counts form the tabs labeled "AIAN," "Asian," "NHPI," and "Two or more races" and divided by the total (AIAN =

American Indian or Alaska Native, NHPI = Native Hawaiian or Pacific Islander). In Connect, I created the "Other" category manually by grouping various (mostly Asian) categories to match their default. For ethnicity (Hispanic or not), I used the 2022 counts in the relevant sections of the "Total" tab and divided by the total.

Census Region

Data for census region were also from the U.S. Census Bureau (2024, November 25). I used a table titled "Estimates of the Total Resident Population and Resident Population Age 18 Years and Older for the United States, Regions, States, District of Columbia, and Puerto Rico: July 1, 2023 (SCPRC-EST2023-18+POP)." The data are for 2023. I used the numbers for "Resident Population Age 18 Years and Older" at the top of the table and divided by the total. In Connect, I grouped individual states (and the District of Columbia) into the four census regions. I reluctantly excluded Puerto Rico because the U.S. Census Bureau does not assign it to a region. Information on the makeup of the regions is widely available, including at https://www2.census.gov/geo/pdfs/maps-data/maps/reference/us_regdiv.pdf.

Personal Income

Data for personal income were from the U.S. Census Bureau (2024, August 16). I used the first table (for "Both Sexes/Total Work Experience/18 Years and Over") on the page tilted "PINC-02. Marital Status-People 18 Years Old and Over, by Total Money Income, Work Experience, Age, Race, and Hispanic Origin." The data are from 2022. I created three income categories that were somewhat equal in size and could be matched to the categories on Connect. I included those without income in the lowest category.

Education

Data for education were from the U.S. Census Bureau (2023, February 16). I used a table titled "Table 1: Educational Attainment of the Population 18 Years and Over, by Age, Sex, Race, and Hispanic Origin: 2022 (ALL RACES)." The data are from 2022. I used the first row of data, for "Both Sexes/18 years and over." I aggregated counts into three categories that I could match reasonably well by aggregating categories on Connect.

Sampling on Connect

Data collection began on Wednesday, May 29, 2024. The target sample size was 2,214 (Connect's stated maximum for census-matched samples), with quotas for the seven demographic variables discussed above.

By Saturday (day 4, n = 1,787, with 427 surveys remaining to be collected), the quotas for the two higher education categories had been met, and data collection had slowed to a crawl. Some other quotas had also been met (including the race categories "Black or African American" and "Other"), but education seemed the likely culprit. Rather than use Connect's "Speed up" button, which would have relaxed the quotas for all targeted variables, I followed Connect's advice to close the survey, clone it, and launch the clone without the education quotas. More precisely, the clone had one large education category that included all responses except "Prefer not to say." For the other targeted demographic variables, I set the target for each category to the number not yet collected for that category. For example, 807 men out of the target of 1,085 had responded, so I set the new target to 1,085 – 807 = 278. If a target had already been met, I excluded the category from the cloned survey.

By Monday (day 6, n = 2,132, with 82 surveys remaining to be collected), data collection had slowed again. Many quotas had been reached, and the primary group remaining to be surveyed

was White men aged 60–99. I closed the survey, cloned it, and launched the clone with those restrictions. Attempting to use ethnicity (Hispanic or not) led to a message the survey was not feasible, so I dropped quotas for that variable. I also dropped quotas for region and personal income, which each had two categories remaining to be filled. I continued to exclude Puerto Rico and "Prefer not to say" responses.

Data collection continued to be slow. On Wednesday (day 8, n = 2,176, with 38 surveys remaining to be collected), I again closed the survey, cloned it, and launched the clone. I allowed any age (not just 60–99) but still restricted the sample to White men. Data collection was complete within about 20 minutes.

This process guaranteed that the quotas would be filled exactly for gender and race, but not for the other demographic variables. However, because most of the changes noted above occurred very late in the survey process, the initial sample of 2,220 (2,214 + 6 additional respondents who had completed the survey but "timed out" before submitting it to Connect) was very similar to the U.S. Census targets on most demographic variables (see Table S4). The primary exceptions were education and (to a lesser extent) age.

Participant Exclusions

In the Qualtrics settings, I turned on "Prevent multiple submissions," "Bot detection," and "Relevant ID" (to detect fraudulent responses), and "Anonymize responses" (to avoid recording IP addresses, location data, and contact data).

The initial Qualtrics file included 2,255 participants. I first eliminated 35 participants who did not finish the survey (Finished = 0). They either returned their survey in Connect (i.e., they opted out) or they "timed out" because they took more than 20 minutes.

I consider the 2,220 participants who finished the survey to be the initial sample (but see below for a clarification). This number included the target sample in Connect (2,214) plus an additional six participants who completed the survey (Finished = 1 or Progress = 100) but who timed out or had "no code" and thus did not submit the job to Connect for payment. I paid five of these participants via Connect's bonus feature. I did not pay the sixth participant, who had skipped several questions. I also did not pay 41 other participants whom I rejected on Connect (Connect encourages the rejection of participants who provide poor quality data). Specifically, I rejected participants who finished the entire experiment in less than 30 seconds or who made more than three errors (e.g., skipping a question) in eight questions. The rejection criteria for Connect were separate from and more permissive than the preregistered exclusion criteria described below. All participants who were rejected on Connect were later excluded from the analyses, but not all participants who were excluded from the analyses were rejected on Connect. Rejections on Connect did not directly affect the sample size, which remained at 2,220.

Before excluding any other participants, I merged the Qualtrics data with the demographic data provided by Connect. I matched participants' data according to the study-specific participant IDs provided by Connect in both files.

The overall sample of 2,220 included 277 participants in two conditions of a different experiment (preregistered at https://aspredicted.org/hym6-cnd4.pdf. I had run the two experiments simultaneously to make more efficient use of participants in cells related to the standard framing effect (G4 and L6). Excluding participants in the other experiment left 1,943 participants in the replication experiment. I consider 1,943 to be the initial sample size *for the replication experiment* (all participants who finished the replication experiment). See Table S4 for demographic information for these participants.

Following the preregistered criteria, I excluded participants who were flagged by Qualtrics as fraudulent or duplicates (94 participants with RecapchaScore < 0.5, RelevantIDDuplicateScore ≥ 75, or RelevantIDFraudScore ≥ 30). Next, I excluded participants whose completion time was more than 2.5 *SD*s away from the mean completion time (after normalizing the distribution with a Box-Cox transformation; 17 participants with completion times that were too long and 15 participants with completion times that were too short) or whose completion time was less than 45 seconds (0 participants). Note that those excluded for taking too long included the six participants whom I had added at the beginning even though they had “timed out” (but, hey, I excluded them according to a *preregistered* criterion; LOL). Finally, I excluded participants who skipped more than one of the eight choice and rating questions (40 participants) or answered any pair of choice and rating questions inconsistently (e.g., choosing Program A but preferring Program B; 80 participants). I retained three participants who mentioned framing in response to a debriefing question; they did not fit any preregistered exclusion criterion. The final sample size was 1,697, which was rather close to the target of 1,680. See Table S4 for demographic information for the final sample.

Example Scenarios and Options in the Four Domains

The disease scenario was based on the original disease problem (Tversky & Kahneman, 1981). The drought, investment, and wildfire scenarios were based on problems in Peters and Levin (2008). An example screenshot is shown in Figure S1.

The wording for problems with completely described options in both frames (cells G5 and L5 in Table 1 of the main text) is shown below. Other versions of the problems omitted the good or bad aspect of the certain option, the good or bad aspect of the risky option, or one (possibly different) aspect of each option. When relevant, the good and bad aspects could also be

Figure S1

A Screenshot Showing a Choice in the Gain Frame of the Disease Domain, With Both Options Described Completely (Like Cell G5 in Table 1 of the Main Text but With Different Numbers).

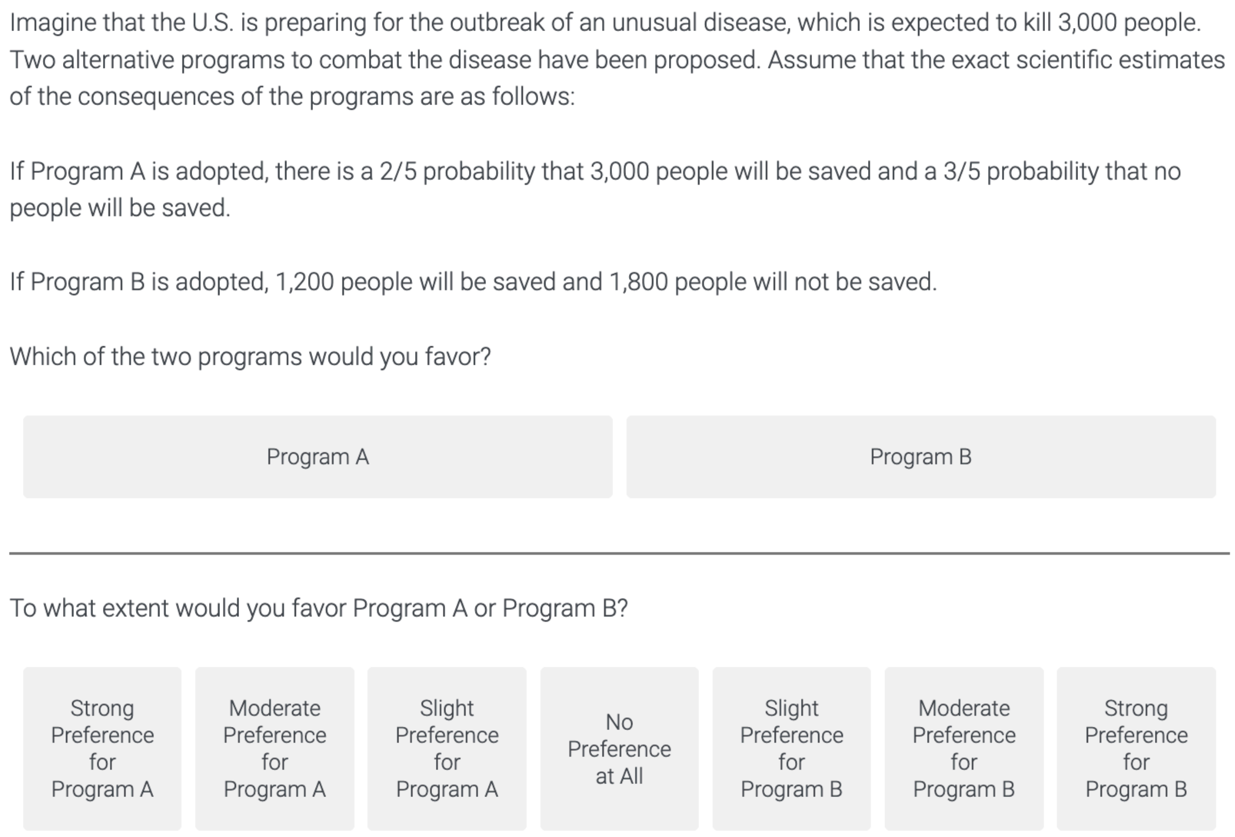

mentioned in the opposite order, with the bad aspect coming first. The cover story, the included aspects, and (when relevant) the order of aspects were matched in the gain and loss frames.

Disease

Gain Frame

Imagine that the U.S. is preparing for the outbreak of an unusual disease, which is expected to kill 3,000 people. Two alternative programs to combat the disease have been proposed. Assume that the exact scientific estimates of the consequences of the programs are as follows:

If Program A is adopted, 1,200 people will be saved and 1,800 people will not be saved.

If Program B is adopted, there is a 2/5 probability that 3,000 people will be saved and a 3/5 probability that no people will be saved.

Loss frame

If Program A is adopted, 1,200 people will not die and 1,800 people will die.

If Program B is adopted, there is a 2/5 probability that no people will die and a 3/5 probability that 3,000 people will die.

Drought

Gain Frame

Imagine that a severe drought is foreseen to hit the state of Missouri next summer. The drought will cause the destruction of 24,000 acres of crops. Two programs of water supply have been proposed:

If Program A is adopted, 12,000 acres of crops will be saved and 12,000 acres of crops will not be saved.

If Program B is adopted, there is a 50% chance that 24,000 acres of crops will be saved and a 50% chance that no acres of crops will be saved.

Loss Frame

If Program A is adopted, 12,000 acres of crops will not be lost and 12,000 acres of crops will be lost.

If Program B is adopted, there is a 50% chance that no acres of crops will be lost and a 50% chance that 24,000 acres of crops will be lost.

Investment

Gain Frame

Suppose that you have invested $15,000 in the stock of a company that just filed for bankruptcy. The company has proposed two alternatives to reduce the effect on your investment:

If Program A is adopted, $5,000 will be saved and $10,000 will not be saved.

If Program B is adopted, there is a one out of three chance that $15,000 will be saved and a two out of three chance that no money will be saved.

Loss Frame

If Program A is adopted, $5,000 will not be lost and $10,000 will be lost.

If Program B is adopted, there is a one out of three chance that no money will be lost and a two out of three chance that $15,000 will be lost.

Wildfire

Gain Frame

Imagine that the wildfire season is about to start and an old-growth forest in the Northwest of the U.S. will be affected. This forest is home to 7,200 animals that are endangered by the fire. Two programs have been proposed to protect the animals:

If Program A is adopted, 1,800 animals will be saved and 5,400 animals will not be saved.

If Program B is adopted, there is a one-fourth probability that 7,200 animals will be saved and a three-fourths probability that no animals will be saved.

Loss Frame

If Program A is adopted, 1,800 animals will not die and 5,400 animals will die.

If Program B is adopted, there is a one-fourth probability that no animals will die and a three-fourths probability that 7,200 animals will die.

Power Analyses

Prior to data collection (indeed, prior to requesting funding), I conducted simulation-based power analyses using the procedures described by Kumle et al. (2021, Scenario 3). Those procedures required (a) the experimental design and target sample size, (b) specified slopes for the effects of interest, and (c) estimates of some other parameters of the model (in my case, the intercept and its random variance). These analyses, in conjunction with practical survey-design issues (e.g., how to easily generate unequal cell sizes in Qualtrics), helped us determine the design and target sample size. I am aware that these analyses omit various uncertainties, such as that associated with effect-size heterogeneity (De Boeck et al., 2024; Kenny & Judd, 2019; Pawel & Held, 2020; Pek et al., 2024).

I conducted additional power analyses after the data were collected, for three reasons: to increase the precision of some estimates with more simulation runs, to generate power estimates for less essential comparisons, and to investigate the effect of converting a standardized effect-size measure to an unstandardized regression slope in a way that could be more appropriate for mixed models. None of these refinements used the new data in any way. I think the results are informative even if they played no role in the design of the study.

Of the results reported below, the most important power estimates are for the comparison involving completely described options (G5 vs. L5) and for the primary model that used all the data.

Converting a Standardized Effect-Size Measure to an Unstandardized Slope

Kumle et al.'s (2021) procedures require slopes rather than standardized effect sizes as input. For *Frame*, I chose slopes corresponding to medium, small, and very small effect sizes (Cohen's *d*s = 0.50, 0.20, and 0.10, respectively). Following DeKay and Dou (2024), I multiplied these *d*

values by $\pi/\sqrt{3} \approx 1.81$ to yield unstandardized logistic regression slopes, which are logs of odds ratios. This conversion (often in the opposite direction) is recommended for meta-analysis (Borenstein et al., 2009), but other ratios have also been suggested (e.g., 1.65; see Sanchez-Mecca et al., 2003).

The logic behind the conversion is as follows. Cohen's *d* is defined as the difference between the means in two conditions divided by the pooled standard deviation, which is an estimate of the within-condition standard deviation. In a logistic regression model with a dichotomous predictor like *Frame*, coded so that there is a one-unit difference between conditions, the coefficient of the predictor gives the numerator. The denominator is given by the standard deviation of the standard logistic distribution, $\pi/\sqrt{3} \approx 1.81$, so $d = b/(\pi/\sqrt{3})$. This expression for *d* is in the logit metric, not the probability metric. The conversion from *d* to *b* is then:

$$b = d \times \pi/\sqrt{3}. \tag{S17}$$

However, the conversion above is typically used for regular (not mixed-effects) logistic regression, and its application to mixed-effects models is questionable because it omits the variance of the random intercept. This seems important, because the inclusion of random effects in logistic regression models is known to increase the coefficients: When the variance is partitioned into level-1 and level-2 components, the entire model is scaled up to maintain a constant level-1 error variance (see, e.g., Snijders & Bosker, 2012, pp. 307–309). Kumle et al. (2021) did not address the relationship between standardized effect-size measures and unstandardized slopes in complicated mixed models, other than to say that it is "far from trivial" (p. 2533).

It is perhaps useful to see where an estimate of the within-condition standard deviation in a two-level mixed-effects model might come from. In the present case, the logistic regression equation for a simple two-cell comparison with only a random intercept is as follows:

$$\text{logit}[P(Risky)_{hi}] = y_{hi} = \gamma_{00} + u_{0i} + \beta Frame_i + \varepsilon_{hi}. \quad \text{(S18)}$$

In this equation, γ_{00} is the global intercept and u_{0i} is the random effect for the intercept, with a subscript *i* for participants and variance τ^2. $Frame_i$ has a value of –0.5 or 0.5, depending on the participant *i*. For completeness, the subscript *h* is for *Domain*. Because I did not control for *Domain* in most of the models, any variation in responses across domains is absorbed into the level-1 (within-participant) error term, ε_{hi}, which has a standard logistic distribution with variance $\pi^2/3$.

In either *Frame* condition, the within-condition variance is the variance of Equation S18:

$$Var(y_{hi,Frame}) = \tau^2 + \pi^2/3\,. \quad \text{(S19)}$$

The within-condition standard deviation is:

$$SD(y_{hi,Frame}) = \sqrt{\tau^2 + \pi^2/3}\,. \quad \text{(S20)}$$

A measure analogous to Cohen's *d*, still in the logit metric, is:

$$d = b/\sqrt{\tau^2 + \pi^2/3}\,. \quad \text{(S21)}$$

And the conversion from *d* to *b* is:

$$b = d \times \sqrt{\tau^2 + \pi^2/3}\,. \quad \text{(S22)}$$

In practice, the estimate of τ^2 can be used. Compared to Equation S17, Equation S22 yields larger slopes, which in turn lead to greater estimated power. For models with more random effects (e.g., crossed or nested structures, random slopes), analogous logic (estimating the within-condition standard deviation) would lead to the inclusion of additional variance or covariance terms, as in the case of linear mixed-effects models (Westfall et al., 2014). I do not

claim that Equations S21 and S22 are statistically or psychometrically correct (there could well be complications), but the approach seems better than ignoring the random effects.

For *Frame*, I chose slopes corresponding to medium, small, and very small effect sizes (*d*s = 0.50, 0.20, and 0.10, respectively). In the first approach (Equation S17), these correspond to slopes of 0.907, 0.363, and 0.181, respectively. In the second approach (Equation S22), the corresponding slopes are larger, but how much larger depends on the estimate of the intercept variance in the appropriate model reported by DeKay and Dou (2024).

Power for Choices With Completely Described Options

For the framing effect in choices with completely described options (L5–G5), the target sample size was 480 participants (240 in each cell).

I first created artificial data for 480 participants, equally split between the two frames. There were four replications for each participant (for the four domains), yielding a total of 1.920 cases. There were no dependent-variable values (for choices) in these data. I then used the simr package in R (Green & MacLeod, 2016) to create a GLMM (generalized linear mixed model) object with parameter values taken from the corresponding model based on DeKay and Dou's (2024) combined sample. Finally, I used the mixedpower package (Kumle et al., 2021) to simulate thousands of data sets (replicates), test the significance of the effects of interest in each simulated data set, and compute power for each effect as the proportion of data sets with significant results. A useful feature of mixedpower is that it can report power not only for the parameter values used to construct the GLMM (called "databased" in the output), but also for a different set of parameter values that often represent smaller effect sizes. This different parameter set is called the "SESOI" (smallest effect size of interest) set. I used the SESOI feature to assess power for the slopes of interest. At least 1,000 runs (simulated data sets or replicates) are recommended,

but using more runs yields more precise estimates. I almost always used 10,000 runs. Depending on the details, this can take from a few minutes to several hours on an otherwise fast personal computer with multiple cores.

For *Frame*, I used two sets of slopes from the two approaches above. In Set 1 (based on Equation S17), I used slopes of 0.907 (medium), 0.363 (small), and 0.181 (very small). In Set 2 (based on Equation S22), I used the estimated intercept variance from the corresponding model based on DeKay and Dou's (2024) combined sample (0.702). The resulting slopes were 0.999 (medium), 0.400 (small), and 0.200 (very small).

Results for the slopes in Set 1 are shown below. Estimated power for detecting a small effect of *Frame* was .84.

```
Results based on 10,000 runs
#          480      mode effect
#Frame       1 databased  Frame slope = 0.844 (DeKay & Dou, BOTH samples)
#Frame1      1     SESOI  Frame slope = 0.907 (medium, set 1)
#Frame  0.8353     SESOI  Frame slope = 0.363 (small, set 1)
#Frame  0.4728     SESOI  Frame slope = 0.181 (very small, set 1)
```

For Set 1, obtaining power = .80 for a very small effect of *Frame* would require more than 1,600 participants (three sample sizes are shown below).

```
#Results based on 5,000 runs
#        1600   1650   1700  mode effect
#Frame 0.7902 0.8076 0.8162 SESOI  Frame slope = 0.181 (very small, set 1)
```

For Set 2, estimated power for detecting a small effect of *Frame* was .89.

```
#Results based on 10,000 runs
#         480      mode effect
#Frame      1     SESOI  Frame slope = 0.999 (medium, set 2)
#Frame 0.8917     SESOI  Frame slope = 0.400 (small, set 2)
#Frame 0.5078     SESOI  Frame slope = 0.200 (very small, set 2)
```

Power for Other Matched Comparisons

For most of the other matched comparisons (e.g., L1–G1, L2–G2), the target sample size was 120 participants (60 in each cell). For these power analyses, I used the average parameter estimates from eight matched comparisons (all except L5–G5) from DeKay and Dou's (2024) combined sample. Reflecting the larger intercept variance (1.890), the resulting slopes in Set 2 were 1.138 (medium), 0.455 (small), and 0.228 (very small).

For Set 1, estimated power was .74 for detecting a medium effect of *Frame*, but only .20 for detecting a small effect.

```
#Results based on 10,000 runs
#          120      mode effect
#Frame  0.7600 databased  Frame slope = 0.934 (DeKay & Dou, BOTH samples)
#Frame1 0.7434     SESOI  Frame slope = 0.907 (medium, set 1)
#Frame  0.1975     SESOI  Frame slope = 0.363 (small, set 1)
#Frame  0.0917     SESOI  Frame slope = 0.181 (very small, set 1)
```

For Set 2, estimated power was .90 for detecting a medium effect of *Frame*, but only .27 for detecting a small effect.

```
#Results based on 10,000 runs
#         120  mode effect
#Frame 0.9018 SESOI  Frame slope = 1.138 (medium, set 2)
#Frame 0.2652 SESOI  Frame slope = 0.455 (small, set 2)
#Frame 0.1164 SESOI  Frame slope = 0.228 (very small, set 2)
```

It is important to remember that comparisons involving only 120 participants are based on only 7% of the full sample. These power estimates are less important than those for the framing effect in choices with completely described options (L5–G5, above) and the primary model (below).

Power for the Standard Framing Effect

For the standard framing effect (L6–G4), the target sample size was 120 participants (60 in each cell). I used parameter estimates from the corresponding analysis in DeKay and Dou's (2024) combined sample. Reflecting the estimated intercept variance (1.932), the resulting slopes in Set 2 were 1.143 (medium), 0.457 (small), and 0.229 (very small).

For Set 1, estimated power was .80 for detecting a medium effect of *Frame*, but only .34 for detecting a small effect. However, I was not very concerned about the possibility of a small effect for the traditional comparison.

```
#Results based on 10,000 runs
#          120      mode effect
#Frame  0.9968 databased  Frame slope = 1.638 (DeKay & Dou, BOTH samples)
#Frame1 0.8044     SESOI  Frame slope = 0.907 (medium, set 1)
#Frame  0.3357     SESOI  Frame slope = 0.363 (small, set 1)
#Frame  0.2476.    SESOI  Frame slope = 0.181 (very small, set 1)
```

For Set 2, estimated power was .93 for detecting a medium effect of *Frame*, but only .40 for detecting a small effect.

```
#Results based on 10,000 runs
#         120  mode effect
#Frame 0.9306 SESOI  Frame slope = 1.143 (medium, set 2)
#Frame 0.4049 SESOI  Frame slope = 0.457 (small, set 2)
#Frame 0.2705 SESOI  Frame slope = 0.229 (very small, set 2)
```

Power for the Primary Model Using the Full Design

The experimental design is described in the main text. The target sample size was 1,680 participants, with targets for individual cells of 60 participants (12 cells), 120 participants (4 cells), or 240 participants (2 cells). There were four replications for each participant (for the four domains), yielding a target of 6,720 observations.

To estimate power for this design, I followed procedures analogous to those for the two-cell comparisons above. I first created artificial data for the full design and sample size, with the corresponding values of *ValenceDiff* and *Frame* for each observation. There were no dependent-variable values (for choices) in these data. I then used the simr package in R (Green & MacLeod, 2016) to create a GLMM object with parameter values from DeKay and Dou's (2024) primary model for their combined sample. Finally, I used Kumle et al.'s (2021) mixedpower package to estimate power for different effect sizes.

The Set 1 slopes were the same as those used above: 0.363 for a small effect and 0.181 for a very small effect. I did not assess power for a medium effect because I knew power would be very high even for small effects and because the simulations for the full model with a large sample size take a long time to run (e.g., overnight for 10,000 runs). Reflecting the intercept variance (1.954), the Set 2 slopes were 0.458 for a small effect and 0.229 for a very small effect. I used these slopes for *ValenceDiff* and for *Frame* (where the slope gives the difference between conditions). For the interaction between *ValenceDiff* and *Frame*, I used the estimate from DeKay and Dou's (2024) analysis of both their samples (0.100) because I was not interested in assessing power for detecting that effect.

For Set 1, estimated power was .997 for a very small effect of *ValenceDiff* and .98 for a small effect of *Frame*, but only .52 for a very small effect of *Frame*.

```
#Small effects: Results based on 10,000 runs
#              1680  mode      effect
#ValenceDiff 1.0000 SESOI ValenceDiff slope = 0.363 (small, set 1)
#Frame       0.9783 SESOI       Frame slope = 0.363 (small, set 1)
#Interaction 0.2644 SESOI Interaction slope = 0.100 (DeKay & Dou samples)

#Very small effects: Results based on 10,000 runs
#              1680  mode      effect
#ValenceDiff 0.9973 SESOI ValenceDiff slope = 0.181 (very small, set 1)
#Frame       0.5243 SESOI       Frame slope = 0.181 (very small, set 1)
#Interaction 0.2779 SESOI Interaction slope = 0.100 (DeKay & Dou samples)
```

For Set 2, estimated power was 1.000 for a very small effect of *ValenceDiff* and .999 for a small effect of *Frame*, but only .72 for a very small effect of *Frame*.

```
#Small effects: Results based on 10,000 runs
#              1680  mode      effect
#ValenceDiff 1.0000 SESOI ValenceDiff slope = 0.458 (small, set 2)
#Frame       0.9987 SESOI       Frame slope = 0.458 (small, set 2)
#Interaction 0.2639 SESOI Interaction slope = 0.100 (DeKay & Dou samples)

#Very small effects: Results based on 10,000 runs
#              1680  mode      effect
#ValenceDiff 1.0000 SESOI ValenceDiff slope = 0.229 (very small, set 2)
#Frame       0.7185 SESOI       Frame slope = 0.229 (very small, set 2)
#Interaction 0.2798 SESOI Interaction slope = 0.100 (DeKay & Dou samples)
```

Robustness Checks for the Primary Interval-Scaled EVA Model

In this section and later sections of this Supplement, I report a variety of additional logistic and linear regressions. The goal was to assess the robustness of the primary findings reported in the main text. As anticipated, the effects in Figures 1 and 2 and the results for model comparisons in Table 5 appear to be very robust.

Separate Analyses for Each Domain, Domain Position, and Option Order

I assessed whether the results in the top panel of Figure 1 in the main text held in each of the four domains, each of the four domain positions (first, second, third, and fourth), and each of the two option orders (certain first, risky first).

Domain

In accordance with the preregistration, I conducted a separate logistic regression for each domain. Because there was only one choice per participant in each domain, these were regular rather than mixed-effects logistic regressions. The effect of *ValenceDiff* was significant in each of the four domains (all bs ≥ 0.56, all ps $< .001$), as was the effect of *Frame* (all bs ≥ 0.46, all ps

< .001). The interaction between the two predictors was never significant (all *p*s ≥ .108). The full results appear in Table S5.

Table S5

Logistic Regression Results for Each of the Four Domains

Predictor	Domain			
	Disease	Drought	Investment	Wildfire
Constant	–0.44***	–0.67***	–0.77***	–0.70***
Valence difference	0.56***	0.63***	0.75***	0.74***
Frame	0.78***	0.52***	0.46***	0.51***
Interaction	0.13	–0.02	0.09	0.17

****p* < .001.

Comparisons of models for the entire dataset indicated that the effect of *ValenceDiff* differed significantly across domains (for the interaction, $\chi^2(3)$ = 12.78, *p* = .005) and that the effect of *Frame* differed significantly across domains (for the interaction, $\chi^2(3)$ = 7.91, *p* = .048). These analyses were not preregistered. See the R output for details.

Domain Position

Domains were randomly ordered for each participant. Domain position indicates the first, second, third, or fourth domain considered. In accordance with the preregistration, I conducted a separate logistic regression for each domain position. The effect of *ValenceDiff* was significant in each of the four domain positions (all *b*s ≥ 0.63, all *p*s < .001), as was the effect of *Frame* (all *b*s ≥ 0.48, all *p*s < .001). The interaction between the two predictors was never significant (all *p*s ≥ .101). The full results appear in Table S6.

A model for the entire dataset indicated that the effects of *ValenceDiff* and *Frame* did not differ significantly as function of domain position (coded linearly; for the interactions, both *p*s ≥ .102). This analysis was not preregistered. See the R output for details.

Table S6

Logistic Regression Results for Each of the Four Domain Positions

	Domain position			
Predictor	First	Second	Third	Fourth
Constant	–0.78***	–0.69***	–0.51***	–0.58***
Valence difference	0.70***	0.70***	0.63***	0.64***
Frame	0.48***	0.56***	0.60***	0.67***
Interaction	0.04	0.00	0.16	0.13

****p* < .001.

Option Order

I also conducted a separate mixed-effects logistic regression for each option order (certain option first, risky option first). These analyses were not preregistered. The effect of *ValenceDiff* was significant in each option order (both bs ≥ 0.84, both ps < .001), as was the effect of *Frame* (both bs ≥ 0.68, both ps < .001). The interaction between the two predictors was significant when the certain option was listed first ($p = .019$) but not when the risky option was listed first ($p = .780$). The full results appear in Table S7.

Table S7

Mixed-Effects Logistic Regression Results for Each of the Two Option Orders

	Option order	
Predictor	Certain first	Risky first
Constant	–1.00***	–0.64***
Valence difference	0.89***	0.84***
Frame	0.68***	0.82***
Interaction	0.24*	0.03

**p* < .05. ****p* < .001.

A model for the entire dataset indicated that the effects of *ValenceDiff* and *Frame* did not differ significantly as function of option order (for the interactions, both ps ≥ .475). However, there was a main effect of option order, indicating that participants were more likely to choose

option that was listed first ($p < .001$). This analysis was not preregistered. See the R output for details.

Three-Level Models With Participants Nested Within Microstudies or Conditions

For the mixed-effects models reported so far, I followed the preregistration and DeKay and Dou (2024) in using two-level models with only one random effect: a random intercept for participants. An alternative, arguably better approach is to use three-level models with participants nested withing conditions. There is more than one way to construct such models, depending on the grouping of conditions. One way is to treat matched pairs of conditions as *microstudies*, as in DeKay et al.'s (2022) metastudies approach.

In the first application of this approach, I grouped the 18 cells of the design into nine microstudies corresponding to the nine matched comparisons (e.g., L1–G1, L2–G2), as in the bottom panel of Figure 1 in the main text. The results for three such models appear in the second section of Table S8, with the results of the corresponding two-level models in the first section for comparison. For all models in this table, I report the estimated standard deviations of the random effects. There are (at least) three things to note about the three-level models. First, the random effect for microstudies had nonzero variation in all the models, indicating that the intercept varied across microstudies. For simplicity, I do not report significance tests for the random effects (such tests are often problematic). Second, the BICs were smaller than those for the two-level models, indicating better model performance. Third, the results for the fixed effects were essentially unchanged. In the leftmost model, the interaction between *ValenceDiff* and *Frame* was significant ($p = .042$), but the increase in the coefficient was very small.

Recall from the Method section of the main text that when one or both option descriptions were complete, either the good or bad aspect could be listed first. I used both aspect orders when

Table S8

Results of Two- and Three-Level Mixed-Effects Logistic Regression Models With Participants Nested Within Matched Condition Pairs (Microstudies) or Conditions

	Models with interactions		Models without interactions		Models without Frame	
Model and effect	Estimate	BIC	Estimate	BIC	Estimate	BIC
Two-level models						
EVA (interval-scaled)		7562.6		7556.2		7619.2
Fixed effects						
Constant	–0.83***		–0.84***		–0.84***	
Valence difference	0.87***		0.87***		0.87***	
Frame	0.77***		0.78***			
Interaction	0.12					
Random effects (*SD*)						
Intercept (participants)	1.31		1.31		1.32	
Three-level models with participants nested within nine matched condition pairs (microstudies)						
EVA (interval-scaled)		7541.2		7536.2		7603.4
Fixed effects						
Constant	–0.77***		–0.78***		–0.79***	
Valence difference	0.81***		0.82***		0.81***	
Frame	0.76***		0.77***			
Interaction	0.15*					
Random effects (*SD*)						
Intercept (microstudies)	0.31		0.31		0.30	
Intercept (participants)	1.23		1.23		1.29	
Three-level models with participants nested within 14 matched condition pairs (microstudies)						
EVA (interval-scaled)		**7534.2**		**7529.1**		7596.8
Fixed effects						
Constant	–0.80***		–0.81***		–0.81***	
Valence difference	0.79***		0.79***		0.79***	
Frame	0.76***		0.77***			
Interaction	0.15*					
Random effects (*SD*)						
Intercept (microstudies)	0.33		0.33		0.33	
Intercept (participants)	1.22		1.22		1.28	
Three-level models with participants nested within 28 conditions						
EVA (interval-scaled)		7541.5		7533.7		**7539.5**
Fixed effects						
Constant	–0.81***		–0.81***		–0.81***	
Valence difference	0.80***		0.80***		0.80***	
Frame	0.73***		0.73***			
Interaction	0.15					
Random effects (*SD*)						
Intercept (conditions)	0.34		0.35		0.51	
Intercept (participants)	1.22		1.22		1.21	

Note. BIC = Bayesian information criterion. EVA = explicated valence account. Boldface type indicates the best model (the lowest BIC) of each type (with or without interactions or *Frame*). Results are based on models' predictions for all choices.

**p* < .05. ****p* < .001.

possible, leading to 14 conditions in each frame (14 rather than 18 because neither option was completely described in the four corner cells of each frame). Using 14 matched pairs of conditions yielded 14 microstudies. Results for three-level models with 14 microstudies appear in the third section of Table S8. These models fit better (the BICs were lower), but the results were otherwise very similar to those for nine microstudies.

If one abandons the idea of the pairing of cells or conditions to create microstudies, one can use all 18 cells or all 28 conditions as levels of the random effect. Results for three-level models with 28 conditions appear in the bottom section of Table S8. As before, the results for the fixed effects of *ValenceDiff* and *Frame* remained similar. Interestingly, however, the BICs increased for both models with the *Frame* predictor but decreased for the model without *Frame*. This makes sense. In the latter model, the random effect for condition picked up the variation in response proportions that is attributed to *Frame* in the other two models. This interpretation is consistent with the larger standard deviation for the condition random effect in the model without *Frame*. In the models with *Frame*, on the other hand, the change from 14 levels (microstudies) to 28 levels (conditions) yielded no benefit.

What about the heterogeneity of the framing effect? An appealing feature of the metastudies approach (Baribault et al., 2018; DeKay et al., 2022) is that one can assess the variability in the effect of interest across microstudies (i.e., effect-size heterogeneity, as in meta-analysis) by including a random slope for that effect. However, when I added a random slope for *Frame*, to allow that effect to vary over (nine or 14) microstudies, the resulting models were singular. I suspect this occurred because there was not much variation in the framing effect across microstudies (see, e.g., the similar framing effects for nine matched comparisons in the lower panel of Figure 1 in the main text). I did not try to solve the singularity issue.

Finally, if three-level models with participants nested within microstudies outperform the corresponding two-level models, one could also use three-level models to assess the other theory versions in Table 5 of the main text. I did not pursue this approach for two reasons. First, it would take the modeling even further from the preregistered analyses. Second, finding the "best" model (e.g., with the lowest BIC) was not the goal of assessing the three-level specifications in Table S8. The goal was simply to assess the robustness of the effects of *ValenceDiff* and *Frame* in the primary model. The reported results are more than sufficient for that purpose. It is not difficult to find models with lower BICs than those presented above (e.g., one could try a three-level model that allows different coefficients for the valences of risky options and certain options, and perhaps control for option order as well), but such models seem unlikely to affect the main conclusions.

Framing Effects in All 81 Possible Cell Pairs

With nine cells in gains and nine in losses (see Table 1 in the main text), there are 81 possible combinations of gain and loss cells. Following the preregistration and DeKay and Dou (2024), I computed framing effects in all combinations. To predict the direction and magnitude of these effects, I used DeKay and Dou's equation, $FE_{\text{Pred}} = (ValenceDiff_{\text{Losses}} - ValenceDiff_{\text{Gains}})/2$, which scales to +1 for the standard framing effect. Note that FE_{Pred} is not literally a predicted framing effect but rather a predictor based on interval-scaled EVA. The results appear in Figure S2, which looks nearly identical to the plots for DeKay and Dou's two samples in their Figure 1.

As DeKay and Dou (2024) noted, the direction and magnitude of framing effects can be pushed around (amplified, eliminated, or reversed) by pairing gain and loss cells in different ways. DeKay and Dou suggested that two of the highlighted comparisons, L7–G3 and L2–G8,

Figure S2

Framing Effects for All Possible Comparisons of Gain and Loss Cells

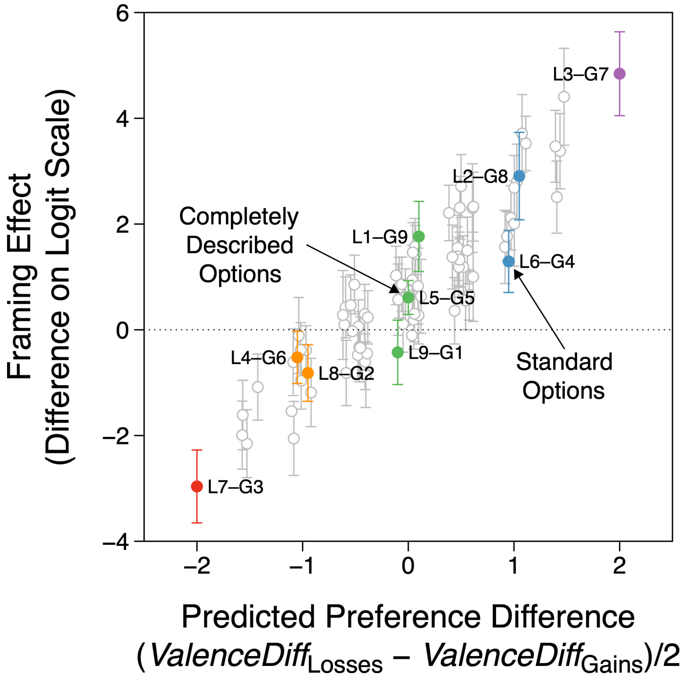

Note. Predicted framing effects on the horizontal axis are from interval-scaled EVA. Framing effects on the vertical axis are the coefficients for *Frame* in separate mixed-effects logistic regression models. A positive framing effect means that the risky option was more popular in the loss frame than in the gain frame. Solid, colored symbols show the standard framing effect and other variants highlighted by DeKay and Dou (2024), most of which were taken from Broniatowski and Reyna (2018). Open, gray symbols show other possible comparisons that rely on recombinations of the same information. Labels correspond to the cells in Tables 1 of the main text. Error bars indicate 95% CIs. To reduce overlap, most points are shifted slightly to the left or right. Based on the predicted preferences in Table 2 in the main text, other theory specifications predict different framing effects for some cell pairs.

might have been novel. However, Stocké (1998) also reported a reversed framing effect in the L7–G3 comparison.

Robustness Checks for Framing Effects in Choices Between Completely Described Options

In this section, I report some additional tests of framing effects in choices between completely described options. Although the simplest test of the framing effect in this matched comparison (L5–G5) was preregistered, the additional analyses reported here were not.

Specifically, I assessed whether the framing effect in choices between completely described options held in each of the four domains, each of the four domain positions (first, second, third, and fourth), and each of the two option orders (certain first, risky first).

Domain

I conducted a separate logistic regression for each domain. Because there was only one choice per participant in each domain, these were regular rather than mixed-effects logistic regressions. The effect of *Frame* was significant at the $p < .05$ level in three of the four domains (for the investment domain, $p = .224$). The results appear in Table S9. In a model for all four domains, the effect of *Frame* did not vary significantly across domains (for the interaction, $\chi^2(3) = 2.18$, $p = .536$). See the R output for details.

Table S9

Logistic Regression Results for Choices Between Completely Described Options in Each of the Four Domains

	Domain			
Predictor	Disease	Drought	Investment	Wildfire
Constant	–0.58***	–0.93***	–1.03***	–0.91***
Frame	0.55**	0.60**	0.25	0.49*

*$p < .05$. **$p < .01$. ***$p < .001$.

Domain Position

I conducted a separate logistic regression for each domain position. The effect of *Frame* was significant at the $p < .05$ level in three of the four domain positions (for the second domain position, $p = .060$). The results appear in Table S10. In a model for all four domains, the effect of *Frame* did not vary significantly across domains (for the interaction, $\chi^2(3) = 2.18$, $p = .536$). See the R output for details.

Table S10

Logistic Regression Results for Choices Between Completely Described Options in Each of the Four Domain Positions

	Domain position			
Predictor	First	Second	Third	Fourth
Constant	–0.92***	–0.92***	–0.74***	–0.87***
Frame	0.44*	0.38†	0.56**	0.52*

†$p < .10$. *$p < .05$. **$p < .01$. ***$p < .001$.

Option Order

I also conducted a separate mixed-effects logistic regression for each option order (certain option first, risky option first). The effect of *Frame* was significant in both option orders (both *p*s ≤ .008). The results appear in Table S11. In a model for both option orders, the effect of *Frame* did not vary significantly in the two orders (for the interaction, $b = 0.25$, $p = .320$). See the R output for details.

Table S11

Mixed-Effects Logistic Regression Results for Choices Between Completely Described Options in Each of the Two Option Orders

	Option order	
Predictor	Certain first	Risky first
Constant	–1.33***	–0.92***
Frame	0.73***	0.55**

**$p < .001$. ***$p < .001$.

Additional Analyses for FTT and EVA, Assuming "Some Not Saved" > "None Saved" and "None Die" > "Some Do Not Die"

In this section, I report more details regarding the new FTT and EVA specifications that treat negations (e.g., "400 people will not be saved") differently than DeKay and Dou's (2024) specifications do.

Fuzzy-Trace Theory

Table S12 shows how well FTT3 (with stronger predicted preferences in cells G3, G7, L3, and L7) and FTT4 (without such amplification) predicted choice proportions in the replication experiment. FTT3 outperformed FTT4 in terms of BIC, but neither version eliminated the residual framing effect (all *p*s ≤ .001). Based on BICs, FTT3 outperformed both FTT1 and FTT2 (see Table 5 of the main text), while FTT4 outperformed FTT1 but not FTT2. The lack of amplified preferences in FTT4 (scores of ±1 rather than ±2; see Table 2 in the main text) seems to be responsible for its weaker performance. However, of all four versions, FTT4 had the smallest coefficients for *Frame*.

The improved performance of FTT3 compared to the versions considered by DeKay and Dou (2024) seems to validate Kühberger and Tanner's (2010) intuition regarding the interpretation of negations in descriptions of the certain option.

The Explicated Valence Account

The results for the reparameterized EVA model in Equation S15 appear in Table S12 on the right side of the middle section. The implied value for the scaling factor *f* was (without rounding) 0.66256/1.08155 = 0.61260. In gains, this value of *f* scaled the original certain-option valences of (+1, 0, –1) to about (+1, +0.39, –0.23). In losses, the original certain-option valences were scaled to about (+0.23, –0.39, –1).

As a check, I used these rescaled valences to compute *RValenceDiff* as in Equation S4. I then used *RValenceDiff* in place of the original *ValenceDiff* in the primary model and in similar models without the interaction and without *Frame*. The results appear in the next-to-last section of Table S12. In each case, I recalculated the BIC values after adding 1 to the parameter count to reflect the separate estimation of *f*. As should be the case, the results for the model without

Table S12

Results of Mixed-Effects Logistic Regression Models for Alternative Specifications of FTT and EVA, Assuming "Some Not Saved" > "None Saved" and "None Die" > "Some Do Not Die"

	Models with interactions		Models without interactions		Models without Frame	
Model and predictor	Coefficient	BIC	Coefficient	BIC	Coefficient	BIC
FTT (interpretation 3)		7564.2		7557.3		7581.0
Constant	–0.84***		–0.84***		–0.83***	
Gist difference	0.88***		0.88***		0.90***	
Frame	0.51***		0.52***			
Interaction	0.11					
FTT (interpretation 4)		7634.1		7627.0		7636.3
Constant	–0.85***		–0.84***		–0.84***	
Gist difference	1.30***		1.30***		1.35***	
Frame	0.39***		0.40***			
Interaction	0.16					
EVA (rescaled, reparameterized)						**7527.4**
Constant					–0.84***	
Shifted valence (risky)					1.08***	
Shifted valence (certain)					–0.66***	
EVA (rescaled, *f* = 0.6126)		<u>**7542.4**</u>		<u>**7535.9**</u>		<u>**7527.4**</u>
Constant	–0.86***		–0.84***		–0.84***	
Valence difference	1.09***		1.09***		1.08***	
Frame	–0.07		–0.06			
Interaction	0.15					
EVA (rescaled, model-specific *f*)	*f* = 0.64	<u>7543.4</u>	*f* = 0.63	<u>7536.5</u>		
Constant	–0.86***		–0.84***			
Valence difference	1.07***		1.08***			
Frame	0.00		–0.01			
Interaction	0.15					

Note. BIC = Bayesian information criterion. FTT = fuzzy-trace theory. EVA = explicated valence account. Boldface type indicates the best model (the lowest BIC) of each type (with or without interactions or *Frame*). Underlined type indicates that the BIC was adjusted upward to account for the separate estimation of the additional parameter *f*. Results are based on models' predictions for all choices.

***$p < .001$.

Frame were identical to the results for the reparameterized model used to estimate *f*. In the other two models, the coefficient for *Frame* was small and nonsignificant, but it was not exactly zero, presumably because the *RValenceDiff* predictor was correlated with *Frame* by design.

Finally, I manually searched values of *f* in increments of 0.01 to find the value that most nearly eliminated the residual framing effect in models with and without the interaction. The

results appear in the last section of Table S12. The values of *f* were 0.64 and 0.63 in the two models, respectively. I again adjusted the BIC values to account for this "estimation" of *f*.

The BICs for all these rescaled EVA models were lower (better) than the BICs of corresponding FTT3 and FTT4 models in the same table. Moreover, they were all lower than the BICs of almost all models in Table 5 of the main text, which includes the versions of EVA and FTT considered by DeKay and Dou (2024).

To summarize these results, rescaling the certain-option valences in accord with Kühberger and Tanner's (2010) intuition regarding the interpretation of negations like "400 people will not be saved" led to big improvements in the fit of FTT and EVA models. Among all models tested in this project, the rescaled EVA models were the only ones to successfully account for the framing effect and thus the only ones to eliminate the *residual* framing effect. Whether the implied valences are reasonable remains open to question. Also, it is possible that other explanations or intuitions could imply codings that lead to similar model performance.

Performance of the New FTT and EVA Models in DeKay and Dou's (2024) Samples

To examine the generality of the above results, I also assessed the performance of FTT3, FTT4, and the rescaled version of EVA in DeKay and Dou's (2024) two samples. None of these analyses was preregistered. Results appear in Table S13 for their CloudReseach sample and in Table S14 for their student sample. For comparison, both tables include results for DeKay and Dou's FTT2 (the better of their two versions of FTT) and their interval-scaled EVA (the best of their three versions of EVA).

As in the replication, FTT3 outperformed FTT2 and FTT4 in terms of BIC in both samples, making it the best of the FTT models. Interestingly, in the models for FTT4, the residual framing

Table S13

Results of Mixed-Effects Logistic Regression Models for Alternative Specifications of FTT and EVA in DeKay and Dou's (2024) CloudResearch Sample

	Models with interactions		Models without interactions		Models without Frame	
Model and predictor	Coefficient	BIC	Coefficient	BIC	Coefficient	BIC
FTT (interpretation 2)		3653.1		3648.2		3663.3
Constant	–1.05***		–1.04***		–1.03***	
Gist difference	1.23***		1.24***		1.29***	
Frame	0.72***		0.75***			
Interaction	0.25†					
FTT (interpretation 3)		*3637.8*		*3631.4*		*3653.7*
Constant	–1.04***		–1.03***		–1.03***	
Gist difference	1.04***		1.05***		1.09***	
Frame	0.83***		0.85***			
Interaction	0.15					
FTT (interpretation 4)		3693.2		3687.4		3396.2
Constant	–1.06***		–1.04***		–1.04***	
Gist difference	1.55***		1.56***		1.64***	
Frame	0.63***		0.65***			
Interaction	0.28					
EVA (interval-scaled, primary)		3629.7		3622.8		3678.0
Constant	–1.03***		–1.03***		–1.03***	
Valence difference	1.04***		1.04***		1.05***	
Frame	1.20***		1.22***			
Interaction	0.13					
EVA (out of sample, *f* = 0.6126)		**3615.5**		**3609.4**		**3603.0**
Constant	–1.06***		–1.03***		–1.03***	
Valence difference	1.28***		1.29***		1.32***	
Frame	0.19		0.21			
Interaction	0.20					
EVA (rescaled, reparameterized)						3609.3
Constant					–1.03***	
Shifted valence (risky)					1.33***	
Shifted valence (certain)					–0.74***	
EVA (rescaled, *f* = 0.55416)		3623.4		3617.5		3609.3
Constant	–1.07***		–1.03***		–1.03***	
Valence difference	1.32***		1.33***		1.33***	
Frame	0.00		0.02			
Interaction	0.21					

Note. BIC = Bayesian information criterion. FTT = fuzzy-trace theory. EVA = explicated valence account. Boldface type indicates the best model (the lowest BIC) in each column. Italic type indicates the lowest-BIC FTT model in each column. Underlined type indicates that the BIC was adjusted upward to account for the separate estimation of the additional parameter *f*. Results are based on models' predictions for all choices.

†$p < .10$. ***$p < .001$.

Table S14

Results of Mixed-Effects Logistic Regression Models for Alternative Specifications of FTT and EVA in DeKay and Dou's (2024) Student Sample

	Models with interactions		Models without interactions		Models without Frame	
Model and predictor	Coefficient	BIC	Coefficient	BIC	Coefficient	BIC
FTT (interpretation 2)		2415.7		2408.1		2407.6
Constant	–0.85***		–0.85***		–0.85***	
Gist difference	0.78***		0.78***		0.80***	
Frame	0.38**		0.39**			
Interaction	0.04					
FTT (interpretation 3)		*2386.8*		*2379.9*		*2380.9*
Constant	–0.85***		–0.85***		–0.85***	
Gist difference	0.70***		0.70***		0.72***	
Frame	0.40**		0.42**			
Interaction	0.09					
FTT (interpretation 4)		2413.8		2406.1		2402.2
Constant	–0.84***		–0.84***		–0.85***	
Gist difference	1.04***		1.04***		1.07***	
Frame	0.29†		0.28†			
Interaction	–0.01					
EVA (interval-scaled, primary)		2392.6		2385.3		2400.1
Constant	–0.85***		–0.85***		–0.86***	
Valence difference	0.68***		0.68***		0.67***	
Frame	0.66***		0.68***			
Interaction	0.06					
EVA (out of sample, *f* = 0.6126)		**2381.0**		**2373.5**		**2365.9**
Constant	–0.86***		–0.85***		–0.85***	
Valence difference	0.86***		0.86***		0.86***	
Frame	–0.00		0.01			
Interaction	0.06					
EVA (rescaled, reparameterized)						2373.0
Constant					–0.85***	
Shifted valence (risky)					0.87***	
Shifted valence (certain)					–0.49***	
EVA (rescaled, *f* = 0.56897)		<u>2387.8</u>		<u>2380.3</u>		<u>2373.0</u>
Constant	–0.86***		–0.85***		–0.85***	
Valence difference	0.88***		0.88***		0.87***	
Frame	–0.10		–0.09			
Interaction	0.05					

Note. BIC = Bayesian information criterion. FTT = fuzzy-trace theory. EVA = explicated valence account. Boldface type indicates the best model (the lowest BIC) in each column. Italic type indicates the lowest-BIC FTT model in each column. Underlined type indicates that the BIC was adjusted upward to account for the separate estimation of the additional parameter *f*. Results are based on models' predictions for all choices.

†$p < .10$. **$p < .01$. ***$p < .001$.

was not quite significant in the smaller student sample (in the models with and without the interaction, respectively, *b*s = 0.29 and 0.28, *p*s = .054 and .053).

For the rescaled version of EVA, I first used the value of *f* from the replication (0.61260) to make out-of-sample predictions in DeKay and Dou's (2024) two samples. In both samples, rescaled EVA outperformed FTT3 and DeKay and Dou's interval-scaled EVA. In addition, the residual framing effect was not significant in any test (all *b*s ≤ 0.21, all *p*s ≥ .183).

I also used the reparameterized version of EVA in Equation S15 to estimate the value of *f* that would eliminate the framing effect in each sample. The unrounded results were *f* = 0.55416 in the CloudReserach sample and *f* = 0.56897 in the student sample. In the CloudResearch sample, the BICs for rescaled EVA remained below those of FTT3 and interval-scaled EVA (as before, the BICs for rescaled EVA were corrected to account for the separate estimation of *f*). Moreover, the residual framing effect remained small and nonsignificant (in the models with and without the interaction, respectively, *b*s = 0.00 and 0.02, *p*s = .992 and .900).

In the student sample, the BICs for rescaled EVA were below those of interval-scaled EVA, but they were ever-so-slightly higher than those for FTT3 (by 1 point or less) when *Frame* was also a predictor. With rescaled EVA, the residual framing effect remained small and nonsignificant (in the models with and without the interaction, respectively, *b*s = –0.10 and –0.09, *p*s = .513 and .549). In contrast, FTT3 did not eliminate the residual framing effect (in the models with and without the interaction, respectively, *b*s = 0.40 and 0.42, *p*s = .006 and .003). Interestingly, when the *Frame* predictor was omitted, rescaled EVA again had a lower BIC than FTT3, as in the CloudResearch sample.

In summary, FTT and EVA models that assume "some not saved" > "none saved" and "none die" > "some do not die" performed better than FTT and EVA models with other assumptions. In

terms of BIC and the elimination of the residual framing effect, the best model in almost every comparison in all three samples (CloudResearch, students, and the replication) was rescaled EVA.

Lower-Bounding Specifications With Different Weights for Interpretations of Certain and Risky Options

The results for two specifications of LBH confirmed the preregistered predictions (see Table S15). All the BICs in Table S15 were smaller than the corresponding BICs for DeKay and Dou's (2024) specifications of FTT and EVA (see Table 5 in the main text). For separate-effects LBH, the coefficient for risky options was almost exactly twice as large as that for certain options. As a result, constraining the ratio to equal 2 improved performance, as indicated by lower the BICs for constrained-effects LBH models. Finally, the residual framing effect remained significant in all LBH models that included *Frame* (all *p*s < .001).

The LBH models also performed well compared to FTT3 and rescaled EVA. Both LBH specifications had lower BICs than the FTT3 in models with the *Frame* predictor, but not in the model without *Frame*. Separate-effects LBH had a trivially lower BIC than rescaled EVA in the model without the interaction, but a higher BIC in the other two models. Constrained-effects LBH had a lower BIC than rescaled EVA in models with the *Frame* predictor, but not in the model without *Frame*.

As noted in the main text, constrained-effects LBH was the only theory version that relied on DeKay and Dou's (2024) data for a parameter estimate or constraint. Aside from the remarkable similarity of the results to DeKay and Dou's results, the take-home message from its performance is not clear.

Table S15

Results of Mixed-Effects Logistic Regression Models for Specifications of LBH with Different Weights for Interpretations of Certain and Risky Options

	Models with interactions		Models without interactions		Models without Frame	
Model and predictor	Coefficient	BIC	Coefficient	BIC	Coefficient	BIC
LBH (separate effects)		7549.9		7534.5		7600.4
Constant	–0.83***		–0.84***		–0.84***	
Interpretation score (risky)	1.13***		1.14***		1.12***	
Interpretation score (certain)	–0.60***		–0.60***		–0.61***	
Frame	0.77***		0.79***			
Interaction (risky)	0.14					
Interaction (certain)	–0.10					
LBH (constrained effects)		**7532.5**		**7525.9**		**7591.9**
Constant	–0.83***		–0.84***		–0.84***	
Interpretation difference (2 × risky – certain)	0.57***		0.58***		0.57***	
Frame	0.77***		0.79***			
Interaction	0.08					

Note. BIC = Bayesian information criterion. LBH = lower-bounding hypothesis. Boldface type indicates the best model (the lowest BIC) of each type (with or without interactions or *Frame*). Results are based on models' predictions for all choices.

***$p < .001$.

The results for separate-effects LBH suggest that interpretations of ambiguous, incompletely described options may play a role in participants' choices. However, the larger coefficient for risky options is more consistent with Kühberger and Tanner's (2010) reasoning than with Mandel's (2014) data regarding lower-bound interpretations, which indicate that such interpretations are more likely for incompletely described certain options than for incompletely described risky options.

Finally, note that the larger effect of risky options is also consistent with rescaled EVA. For example, a scaling factor of 0.61 implies a risky/certain ratio of 1/0.61 = 1.64. In other words, the greater importance of risky options in rescaled EVA is a natural consequence of treating negations in certain options as less extreme than zero outcomes in risky options.

Mixed-Effects Linear Regressions for Predicting Participants' Strength-of-Preference Ratings

In addition to using mixed-effects logistic regressions to predict participants' choices (see Table 5 of the main text), I also used analogous mixed-effects linear regressions to predict participants' strength-of-preference ratings. The results, which appear in Table S16, were essentially identical to those for participants' choices. For example, the orderings of theory versions by their BICs in the columns of Table S16 are nearly identical to those in the corresponding columns of Table 5 of the main text, with the only reversal involving the virtual tie between FTT2 and separate-effects LBH in the rightmost column of Table 5.

Table S16

Results of Mixed-Effects Linear Regression Models for Several Specifications of FTT, EVA, and LBH

Model and predictor	Models with interactions		Models without interactions		Models without Frame	
	Coefficient	BIC	Coefficient	BIC	Coefficient	BIC
FTT (interpretation 1)		24,777.8		24,775.9		24,826.0
Gist difference	0.55***		0.55***		0.55***	
Frame	0.42***		0.43***			
Interaction	0.14**					
FTT (interpretation 2)		24,667.7		24,669.2		*24,683.1*
Gist difference	0.62***		0.62***		0.64***	
Frame	0.26***		0.26***			
Interaction	0.17**					
FTT (interpretation 3)		24,660.5		24,653.4		24,675.2
Gist difference	0.52***		0.52***		0.53***	
Frame	0.30***		0.30***			
Interaction	0.06					
EVA (interval-scaled)		*24,652.6*		*24,647.2*		24,706.2
Valence difference	0.52***		0.52***		0.52***	
Frame	0.44***		0.44***			
Interaction	0.08†					
EVA (original)		24,725.3		24,721.8		24,777.0
Valence difference	0.77***		0.77***		0.76***	
Frame	0.44***		0.44***			
Interaction	0.16*					
EVA (free valence differences)		24,678.4		24,657.9		24,716.7
Positive vs. mixed	0.57***		0.56***		0.57***	
Mixed vs. negative	0.35***		0.35***		0.34***	
Positive vs. negative	1.07***		1.07***		1.07***	
Frame	0.44***		0.44***			
Interaction (pos. vs. mix.)	0.29*					
Interaction (mix. vs. neg.)	0.08					
Interaction (pos. vs. neg.)	0.10					
EVA (rescaled, with *f* = 0.64)		<u>**24,639.2**</u>		<u>24,633.9</u>		<u>**24,625.2**</u>
Valence difference	0.64***		0.64***		0.64***	
Frame	–0.02		–0.02			
Interaction	0.10†					
LBH (separate effects)		24,647.5		**24,633.4**		24,694.5
Interpretation score (risky)	0.66***		0.66***		0.65***	
Interpretation score (certain)	–0.38***		–0.38***		–0.38***	
Frame	0.44***		0.44***			
Interaction (risky)	0.11					
Interaction (certain)	–0.05					

Note. BIC = Bayesian information criterion. FTT = fuzzy-trace theory. EVA = explicated valence account. LBH = lower-bounding hypothesis. Intercepts are omitted (they ranged from –0.52 to –0.50). Italic type indicates the best model (the lowest BIC) of each type (with or without interactions or *Frame*) among the five theory versions considered by DeKay and Dou (2024). Boldface type indicates the best model of each type among all seven theory versions. Underlined type indicates that the BIC was adjusted upward to account for the separate estimation of the additional parameter *f*. Results are based on models' predictions for all choices.

†*p* < .10. **p* < .05. ****p* < .001.

Additional References Not Cited in the Main Text

Baribault, B., Donkin, C., Little, D. R., Trueblood, J. S., Oravecz, Z., von Ravenzwaaij, D., White, C. N., De Boeck, P., & Vandekerckhove, J. (2018). Metastudies for robust tests of theory. *PNAS*, *115*(11), 2607–2612. https://doi.org/10.1073/pnas.1708285114

Green, P., & MacLeod, C. J. (2016). SIMR: An R package for power analysis of generalized linear mixed models by simulation. *Methods in Ecology and Evolution*, *7*(4), 493–498. https://doi.org/10.1111/ 2041-210X.12504

Kenny, D. A., & Judd, C. M. (2019). The unappreciated heterogeneity of effect sizes: Implications for power, precision, planning of research, and replication. *Psychological Methods, 24*(5), 578–589. https://doi.org/10.1037/met0000209

Pawel, S., & Held, L. (2020). Probabilistic forecasting of replication studies. *PLoS ONE, 15*(4), e0231416. https://doi.org/10.1371/journal.pone.0231416

Pek, J., Pitt, M. A., & Wegener, D. T. (2024). Uncertainty limits the use of power analysis. *Journal of Experimental Psychology: General, 153*(4), 1139–1151. https://doi.org/10.1037/xge0001273

Sánchez-Meca, J., Marín-Martínez, F., & Chacón-Moscoso, S. (2003). Effect-size indices for dichotomized outcomes in meta-analysis. *Psychological Methods*, *8*(4), 448–467. https://doi.org/10.1037/1082-989X.8.4.448

Snijders, T. A. B., & Bosker, R. J. (2012). *Multilevel analysis: Introduction to basic and advanced multilevel modeling* (2nd ed.). Sage.

U.S. Census Bureau. (2024, August 16). *PINC-02. Marital Status-People 18 Years Old and Over, by Total Money Income, Work Experience, Age, Race, and Hispanic Origin*. Retrieved

May 22, 2024, from https://www.census.gov/data/tables/time-series/demo/income-poverty/cps-pinc/pinc-02.html

U.S. Census Bureau. (2023, February 16). *Educational attainment in the United States: 2022*. Retrieved May 22, 2024, from https://www.census.gov/data/tables/2022/demo/educational-attainment/cps-detailed-tables.html

U.S. Census Bureau. (2024, November 25). *National population by characteristics: 2020–2024*. Retrieved May 22, 2024, from https://www.census.gov/data/tables/time-series/demo/popest/2020s-national-detail.html#v2023 (The page may have had the dates 2020–2023 when I accessed it.)

Westfall, J., Kenny, D. A., & Judd, C. M. (2014). Statistical power and optimal design in experiments in which samples of participants respond to samples of stimuli. *Journal of Experimental Psychology: General*, *143*(5), 2020–2045. https://doi.org/10.1037/xge0000014
